# The effect and safety of corticosteroid treatment for severe community-acquired pneumonia: a meta-analysis of randomized controlled trials

**DOI:** 10.3389/fmed.2024.1457469

**Published:** 2024-11-06

**Authors:** Yang Chen, Huanming Kuang, Youfeng Zhu, Xing Luo

**Affiliations:** Department of Intensive Care Unit, Guangzhou Red Cross Hospital, Jinan University, Guangzhou, Guangdong, China

**Keywords:** severe community-acquired pneumonia, corticosteroid, hydrocortisone, mortality, meta-analysis

## Abstract

**Background:**

There is ongoing debate on the efficacy and safety of corticosteroid therapy for severe community-acquired pneumonia (sCAP). Our aim was to investigate the safety and therapeutic effectiveness of corticosteroids in the sCAP therapy.

**Methods:**

Electronic databases (Cochrane Library, PubMed, Web of Science and Embase) were searched from inception to January 10, 2024. We examined for randomized controlled studies assessing the effectiveness and safety of corticosteroid therapy in individuals with sCAP. The primary outcome was short-term mortality. Subgroup analyses were carried out according to the corticosteroid type. Additionally, trial sequential analysis (TSA) was carried out.

**Results:**

In total, 11 trials, including 1959 patients, met the predetermined standards and underwent analysis. Overall, our meta-analysis exhibited that corticosteroids may considerably lower short-term mortality when compared to control treatment [6 studies (1,582 patients); odds ratio (OR), 0.65; 95% confidence interval (CI) 0.49–0.88; *p* = 0.005] and C-reactive protein (CRP) levels [5 studies (359 patients); mean difference (MD), −6.97; 95% CI −12.33 to −1.60; *p* = 0.01], but TSA revealed that the sample size needs to be larger. Moreover, we observed that corticosteroids reduced the hospital length of stay [7 studies (999 patients); MD, −3.56; 95% CI, −4.28 to −2.84; *p* < 0.001], need for mechanical ventilation (MV) [7 studies (1,328 patients); OR, 0.60; 95% CI, 0.45–0.79; *p* = 0.001] and MV duration [4 studies (736 patients); MD, −5.62; 95% CI, −7.31 to −3.94; *p* < 0.001], which was in agreement with TSA. However, adverse events, length of hospital and intensive care unit (ICU) stay were not evidently shortened when TSA was utilized. Furthermore, subgroup analysis revealed that all of the above studies benefited from hydrocortisone treatment in comparison to the control group.

**Conclusion:**

Our meta-analysis revealed that corticosteroids, especially hydrocortisone, could decrease the mortality of individuals with sCAP.

**Systematic review registration:**

[https://clinicaltrials.gov/], identifier [CRD42023415555].

## Introduction

Severe community-acquired pneumonia (sCAP) is defined as patients existing community-acquired pneumonia (CAP) and meeting either one main criteria (respiratory failure needing mechanical ventilation or septic shock requiring vasopressor) or three minor criteria (totally nine variables, such as blood urea nitrogen level, respiratory rate, confusion, white blood cell count, etc.) (1, 2). sCAP is the most critically life-threatening form of community-acquired pneumonia and is characterized by rapid progression, critical illness and high morbidity and mortality (3–5). In the United States (US), the estimated number of patients hospitalized with sCAP is 356,326 per year, resulting in 167,474 deaths within 1 year (6). In Europe, the mortality rate of sCAP with invasive mechanical ventilation (MV) in the ICU is 33% (7). Moreover, sCAP has been reported to cause a substantial financial burden on the current medical system (8, 9). Despite advances in life support measures and antimicrobial treatment, the mortality of sCAP remains unacceptably high, suggesting that we need to focus on reducing mortality in other ways (10).

According to previously published studies, sCAP may lead to dysregulated pulmonary and systemic inflammatory responses, which results in deleterious effects and poor prognosis (11–13). Corticosteroids, as inhibitors of inflammation, can act on many cytokines by binding to their specific receptors and decrease the generation of the major inflammatory cytokines (IL-1b, TNFa, IL-6 along with IL-8). Corticosteroids are known to suppress inflammatory responses in specific tissues as well as in the entire body (14, 15). However, the recommendations among international guidelines with regards to the use of corticosteroids in sCAP are divergent. According to a recent European and Latin American guideline, corticosteroids should be considered for use in patients with sCAP if shock occurs (1). The recommendation level is low. In another international guidelines, corticosteroids is only recommended for bacterial sCAP patients, not including viral sCAP (16).

Current evidences report differential results, there is still ongoing debate regarding the use of corticosteroids in sCAP, such as which types of corticosteroids are most effective, and the optimal duration for their use (14, 17, 18). To enhance the clinical prognosis of sCAP, novel therapy options or adjuvant medicines are therefore desperately needed.

The results of two recently published large RCTs (19, 20) remain controversial. One study (19) showed that hydrocortisone could reduce mortality in patients with sCAP. On the other hand, methylprednisolone did not appear to provide any appreciable benefit for individuals with sCAP, according to another research (20). We implemented a meta-analysis of randomized controlled trials (RCTs) to investigate this contentious topic and determine the efficacy of corticosteroids as an adjuvant therapy option in sCAP patients.

### Methods

This research was implemented based on the Preferred Reporting Items for Systematic Reviews and Meta-Analyses (PRISMA) guidelines and was registered in PROSPERO (registration number: CRD42023415555) (21, 22).

### Eligibility criteria

We implemented a comprehensive systematic search of Cochrane Library, PubMed, Web of Science and Embase databases for articles that were published between the creation of the databases and January 10, 2024, with appropriate predefined search terms. The supplemental material contains the specifics of the search method. All peer-reviewed, published trials comparing the safety and effectiveness of corticosteroid therapy to either conventional care or a placebo were found through a comprehensive literature search. The only RCTs considered were those that determined the safety and therapeutic effectiveness of corticosteroids in treating adult sCAP individuals.

The following were the inclusion criteria: (1) population: individuals with sCAP as defined by the included studies; (2) intervention: patients treated with corticosteroids plus conventional therapy; (3) comparison: placebo or standard care; (4) outcomes: all-cause short-term mortality. We defined 28-day (28d) or 30-day (30d) mortality as all-cause short-term mortality in the current meta-analysis. Secondary outcomes are length of hospital and ICU stay, hospital mortality, need for mechanical ventilation (MV), MV time, C-reactive protein (CRP) level and adverse events. (5) Study type: the inclusion criteria were open-ended and included only peer-reviewed RCTs with no limitations on publication date, language, age, sample size, sex, or ethnicity.

We excluded trials that (1) only provided data from *post hoc* analysis; (2) focused on individuals with septic shock or those under the age of 18; (3) were published only as case reports, case series, conference posters, or single-arm studies; (4) did not report relevant outcomes; or (5) were pharmacokinetic studies.

### Data extraction

To assess the possible investigations, two researchers, Yang Chen and Xing Luo, independently examined the abstracts and titles. Arguments were resolved by consensus or by discussion with Youfeng Zhu, the third author. Utilizing a standardized data extraction form, we retrieved the study features (first author, study design, year of publication, number of participants, study site), full study information, controls, interventions, corticosteroid kinds, and outcomes.

### Assessment of risk of bias

With the Cochrane risk-of-bias tool, two researchers (Yang Chen and Huanming Kuang) independently evaluated the risk of bias for all the studies. A third adjudicator (Xing Luo) resolved any disagreements (23).

### Statistical analysis

Review Manager version 5.4 was applied to carry out the statistical analysis. For categorical variables, we computed the OR with a 95% CI, and for continuous variables, we computed the MD with a 95% CI. After determining whether the distribution was skew and normal, continuous variables like the length of hospital and ICU stay, and CRP level—all of which are expressed as medians and interquartile ranges—were transformed into means and standard deviations in accordance with earlier research (24, 25). Random-effects models were applied to pool the data. The I^2^ statistic was utilized to evaluate heterogeneity. Substantial heterogeneity was defined as I^2^ > 50% or *p* < 0.10.

### Subgroup analysis

According to previously published studies, the type and duration of corticosteroid treatment might influence the effect of corticosteroids (17, 18, 26). Therefore, we performed subgroup analyses on the basis of the type and length of corticosteroid therapy.

### Trial sequential analysis

To determine the dependability of our meta-analysis, we employed TSA software (version 0.9.5.10 Beta, Copenhagen Trial Unit, Copenhagen, Denmark) (27). We constructed the O’Brien-Fleming monitoring boundaries with the Lan-DeMets methodology and identified the best informativeness, i.e., an alpha of 0.05, a relative risk reduction of 20%, and a two-sided beta of 0.80. This allowed for the calculation of the required information size (RIS) for primary and secondary outcomes. Next, in order to assess the strength of the evidence, we investigate the relationship between the cumulative Z-curve and the TSA border or RIS.

### Grading the quality of evidence

The quality of evidence for each outcome measure was evaluated utilizing the GRADE methodology (GRADEpro; McMaster University 2014, Hamilton, Canada) (28). The following certainty assessments—indirectness, inconsistency, risk of bias, imprecision, as well as other factors—were applied to lower the quality. Following that, the overall quality of evidence was rated as “high,” “moderate,” “low” as well as “very low.”

## Results

### Search strategy

**Figure 1:**
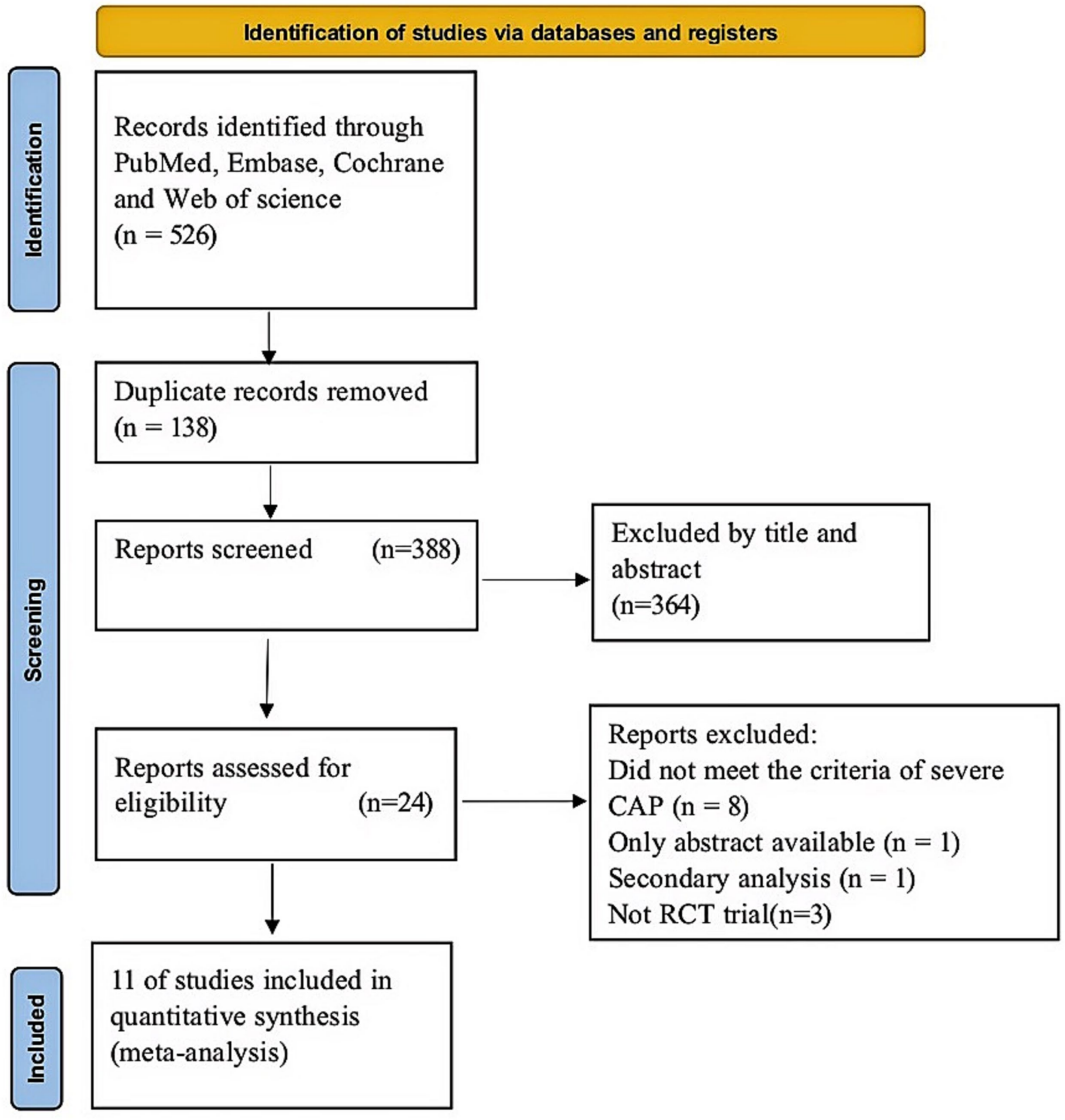
Diagram of the PRISMA flow illustrating the methods for study selection and search.

In the first search, 526 articles were included. Of these, 138 were duplicates and a further 364 studies were excluded through abstract screening. Following the assessment of the full text, 24 studies were eliminated for diverse reasons (Figure 1). Lastly, our study comprised a total of 11 RCTs (19, 20, 29–37). Figure 1 displays the study selection flow diagram.

### Study characteristics

The features of the studies are indicated in Table 1. The analysis comprised a total of 1959 individuals, 988 of whom underwent corticosteroid intervention throughout the study period and 971 of whom received placebo therapy. Nonetheless, the included trials differed in the types of corticosteroids used and the length of the intervention. Six trials administered hydrocortisone (19, 29, 34–37), and five studies administered nonhydrocortisone (20, 29–31, 34). In three trials, patients were given corticosteroids for a period of less than or equal to 5 days (20, 29, 36), and eight trials administered corticosteroids for more than 5 days (19, 20, 31–35, 37).

**Table 1:** Characteristics of the included studies.

Study	Study design	Study site	Sample size	Severe criterion	Population	Interventions	Outcomes
Dequin 2023	Double-blind, randomized placebo-controlled trial	France	400/395	PSI, ICU	All included patients enter the ICU	Hydrocortisone 200 mg iv for 4/8 days, degressive for 8/14 days	Short-term mortality (28/30d), ICU stay, hospital stay, need formechanicalventilation, vasopressors drugs
Meduri 2022	Double-blind, randomized placebo-controlled trial	USA	297/287	ATS, ICU	All included patients enter the ICU	Methylprednisolone 40 mg IV bolus, then 40 mg for 7 days, degressive for 20 days	Short-term mortality (28/30d), In-hospital mortality, ICU stay, hospital stay, need formechanicalventilation, mechanical ventilation time, vasopressors drugs
Ceccato 2016	Double-blind, randomized placebo-controlled trial	Spain	56/50	ATS, ICU, PSI	Adult patients with severe CAP	Methylprednisolo ne 0.5 mg/kg every 12 h for 5 days	In-hospital mortality, ICU stay, hospital stay, need for mechanical ventilation, CRP
Torres 2015	Double-blind, randomized placebo-controlled trial	Spain	55/57	ATS, ICU, PSI	ATS criteria or PSI scores V	Hydrocortisone 0.5 mg/kg every 12 h for 5 days	In-hospital Mortality, ICU stay, hospital stay, need for mechanical ventilation
Nafae 2013	Double-blind, randomized placebo-controlled trial	Egypt	60/20	ATS, ICU	Based on baseline vitals indicating Mean CORB score > 2	Hydrocortisone 200 mg iv bolus, then 10 mg/h for 7 days	In-hospital mortality, ICU stay, hospital stay, need for mechanical ventilation
Ugajin 2013	Double-blind, randomized placebo-controlled trial	Japan	30/71	PSI	All included patients PSI scores V	Corticosteroids (median dosage) = 50 mg/day prednisine shorter than 8 days	Short-term mortality (28/30d), CRP
Fernandez-Serrano 2011	Double-blind, randomized placebo-controlled trial	Spain	23/22	PSI, ICU	Adult patients with ICU were included	Methylprednisolone 200 mg iv bolus, then every 6 h for 3 days, then 20 mg every 12 h for 3 days, then 20 mg for 3 days	In-hospital mortality, ICU stay, hospital stay, need for Mechanical ventilation
El-Ghamrawy 2006	Double-blind, randomized placebo-controlled trial	KSA	17/17	ICU	All included patients enter the ICU	Hydrocortisone, 200 mg IV bolus, then 240 mg for 7 days	In-hospital mortality
Kim 2006	Double-blind, randomized placebo-controlled trial	Korea	13/13	ATS	ATS criteria	Hydrocortisone, 200 mg IV bolus, then 240 mg for7 days	Short-term mortality (28/30d), In-hospital mortality, ICU stay, hospital stay, mechanical ventilation time, CRP
Confalonieri 2005	Double-blind, randomized placebo-controlled trial	Italy	23/23	PSI, ICU	All included patients enter the ICU	Hydrocortisone 200 mg iv bolus, then 10 mg/h for 7 days	Short-term mortality (28/30d), In-hospital mortality, ICU stay, hospital stay, need for mechanical ventilation, mechanical ventilation time, CRP
Marik 1993	Double-blind, randomized placebo-controlled trial	USA	14/16	BTS, ICU	All included patients enter the ICU	Hydrocortisone 10 mg/kg, 1 day	Short-term mortality (28/30d), ICU stay, need for mechanical ventilation

ICU, Intensive care unit; PSI, Pneumonia severity index; ATS, The American Thoracic Society; BTS, British Thoracic Society.

### Quality assessment

In accordance with the risk of bias assessment, two studies were determined to be at high risk of bias (Supplementary Figure S1). There were seven research (29, 31–35, 37) that did not offer approaches for allocation concealment or random sequence generation. The blinding approach may have been violated in four trials, which might have led to an overestimation or underestimation of the magnitude of effect (29, 31, 32, 35). Additionally, six trials (29, 31–33, 35, 37) had an uncertain risk of other bias assigned to them, as these trials have been subject to uncertainty in previous assessments.

### Primary outcome

**Figure 2:**
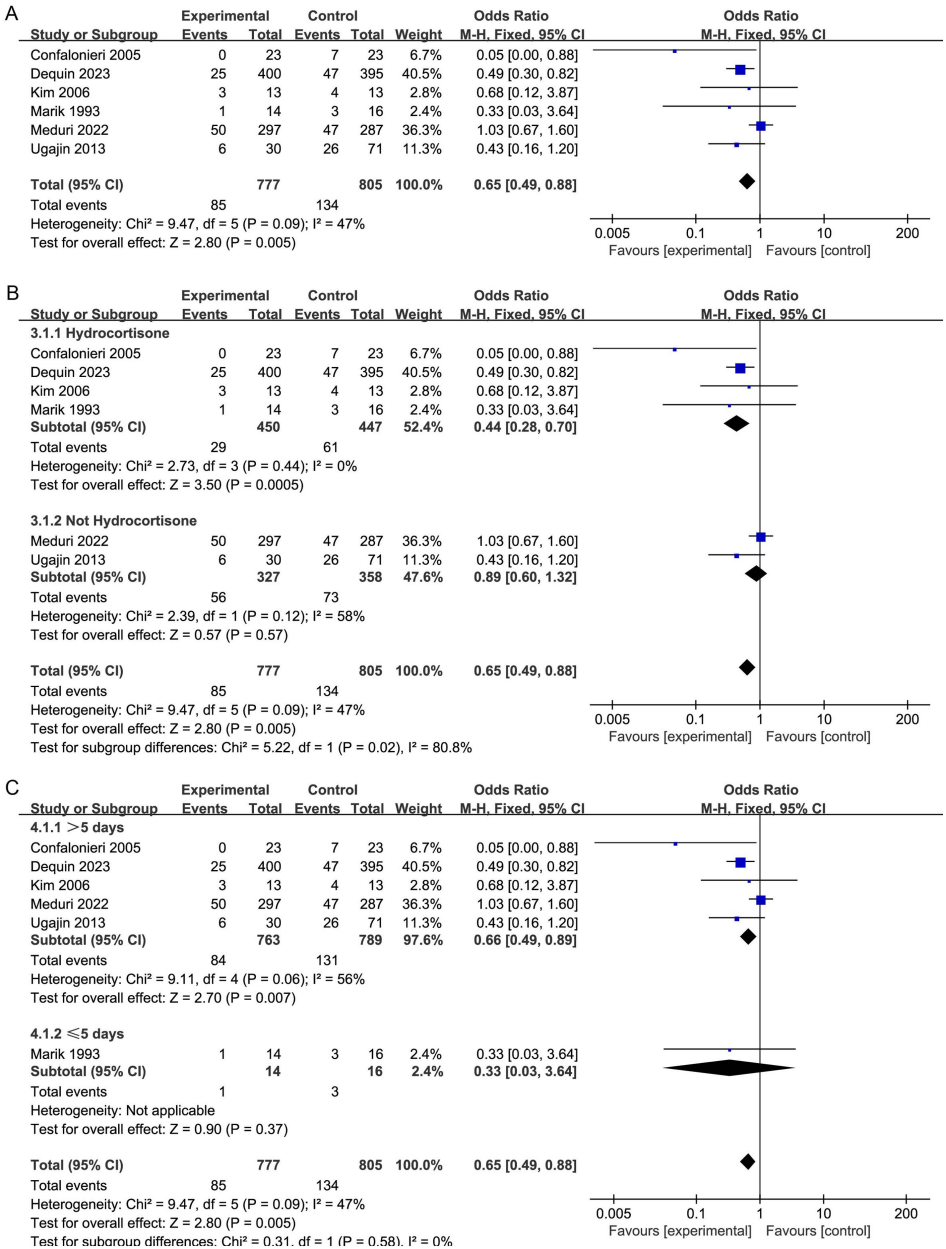
Meta-analysis of duration of hospital stay in individuals taking corticosteroid treatment versus control group **(A)**, subgroup analysis of corticosteroid type **(B)**, and subgroup analysis of duration **(C)**.

Overall, our research displayed that the corticosteroid intervention group had a reduced short-term mortality rate than the control group (OR, 0.65; 95% CI 0.49–0.88; *p* = 0.005, six RCTs (15, 16, 27, 31–33), 1,582 patients, low certainty; Table 2; Figure 2A). However, the TSA-adjusted CIs ranged from 0.40 to 1.06 (Supplementary Figure S2). The cumulative Z-curve crossed the traditional benefits monitoring line, but not the O’Brien-Fleming monitoring line. In addition, it did not cross either the uselessness monitoring line or the RIS, suggesting that currently, there may be false-positive results. Therefore, additional large RCTs are needed to confirm our findings.

**Table 2:** The quality of evidence for each outcome measure was assessed following the GRADE.

Corticosteroid compared to Placebo for severe community-acquired pneumonia						
**Patient or population:** patients with severe community-acquired pneumonia Settings: **Intervention:** Corticosteroid Comparison: Placebo						
Outcomes	Illustrative comparative risks* (95% CI)		Relative effect (95% CI)	No of Participants (studies)	Quality of the evidence (GRADE)	Comments
	Assumed risk	Corresponding risk				
	Placebo	Corticosteroid				
Short-term mortality (28/30)	Study population		OR 0.65 (0.49 to 0.88)	1,582 (6 studies)	⊕ ⊕ ⊝⊝ **low**^1^^,^^2^	
	166 per 1,000	115 per 1,000 (89–149)				
	Moderate					
	246 per 1,000	175 per 1,000 (138–223)				
In-hospital mortality	Study population		OR 0.7 (0.47–1.03)	1,021 (8 studies)	⊕ ⊕ ⊕⊝ moderate^3^	
	133 per 1,000	97 per 1,000 (67–136)				
	Moderate					
	211 per 1,000	158 per 1,000 (112–216)				
Duration of ICU Stay		The mean duration of icu stay in the intervention groups was 0.58 lower (1.19 lower to 0.04 higher)		998 (8 studies)	⊕ ⊕ ⊕⊝ moderate^4^	
Hospital stay		The mean hospital stay in the intervention groups was 3.56 lower (4.28–2.84 lower)		999 (7 studies)	⊕ ⊕ ⊝⊝ low^5^	
Need for mechanical ventilation	Study population		OR 0.6 (0.45–0.79)	1,382 (7 studies)	⊕ ⊕ ⊕⊝ moderate^6^	
	218 per 1,000	143 per 1,000 (111–180)				
	Moderate					
	227 per 1,000	150 per 1,000 (117–188)				
Mechanical ventilation time		The mean mechanical ventilation time in the intervention groups was 5.62 lower (7.31–3.94 lower)		736 (4 studies)	⊕ ⊕ ⊕⊝ moderate^7^	
CRP		The mean crp in the intervention groups was 6.97 lower (12.33–1.6 lower)		359 (5 studies)	⊕⊝⊝⊝ very low^8^^,^^9^	
Gastrointestinal hemorrhage	Study population		OR 0.74 (0.38–1.46)	1,221 (7 studies)	⊕ ⊕ ⊕⊝ moderate^10^	
	31 per 1,000	23 per 1,000 (12–45)				
	Moderate					
	33 per 1,000	25 per 1,000 (13–47)				
Frequency of hyperglycemia requiring treatment	Study population		OR 1.12 (0.58–2.14)	346 (4 studies)	⊕ ⊕ ⊕⊝ moderate^11^	
	116 per 1,000	128 per 1,000 (71–220)				
	Moderate					
	95 per 1,000	105 per 1,000 (57–183)				
Incidence of hospital-acquired infection	Study population		OR 0.84 (0.55–1.28)	1,096 (5 studies)	⊕ ⊕ ⊕⊝ moderate^12^	
	96 per 1,000	82 per 1,000 (55–119)				
	Moderate					
	70 per 1,000	59 per 1,000 (40–88)				

*The basis for the assumed risk (e.g., the median control group risk across studies) is provided in footnotes. The corresponding risk (and its 95% confidence interval) is based on the assumed risk in the comparison group and the relative effect of the intervention (and its 95% CI). CI, Confidence interval; OR, Odds ratio; GRADE Working Group grades of evidence; High quality, Further research is very unlikely to change our confidence in the estimate of effect. Moderate quality, Further research is likely to have an important impact on our confidence in the estimate of effect and may change the estimate. Low quality, Further research is very likely to have an important impact on our confidence in the estimate of effect and is likely to change the estimate. Very low quality, We are very uncertain about the estimate.

1 Does not meet 2/3 low risk.

2 OR, 0.65.

3 95%CI[0.47, 1.03].

4 95%CI[−1.19, 0.04].

5 I^2^ = 91%, unable to explain.

6 OR, 0.60.

7 I^2^ = 68%, unable to explain.

8 I^2^ = 86%, unable to explain.

9 Simple size <400.

10 95%CI[0.38, 1.46].

11 95%CI[0.58, 2.14].

12 95%CI[0.55, 1.28].

Furthermore, our subgroup analysis revealed that the hydrocortisone subgroup resulted in a considerable decrease in the short-term mortality of sCAP patients, and the TSA results were consistent with this result (OR, 0.44; 95% CI, 0.28–0.70; *p* = 0.005; Figure 2B; Supplementary Figure S2). Nevertheless, these benefits were not observed in the nonhydrocortisone subgroup (Figure 2B).

Moreover, the subgroup analysis revealed that corticosteroid therapy longer than 5 days may significantly lower short-term mortality (OR, 0.66; 95% CI 0.49–0.89; *p* = 0.007; Figure 2C).

### Secondary outcomes

#### Hospital mortality

**Figure 3:**
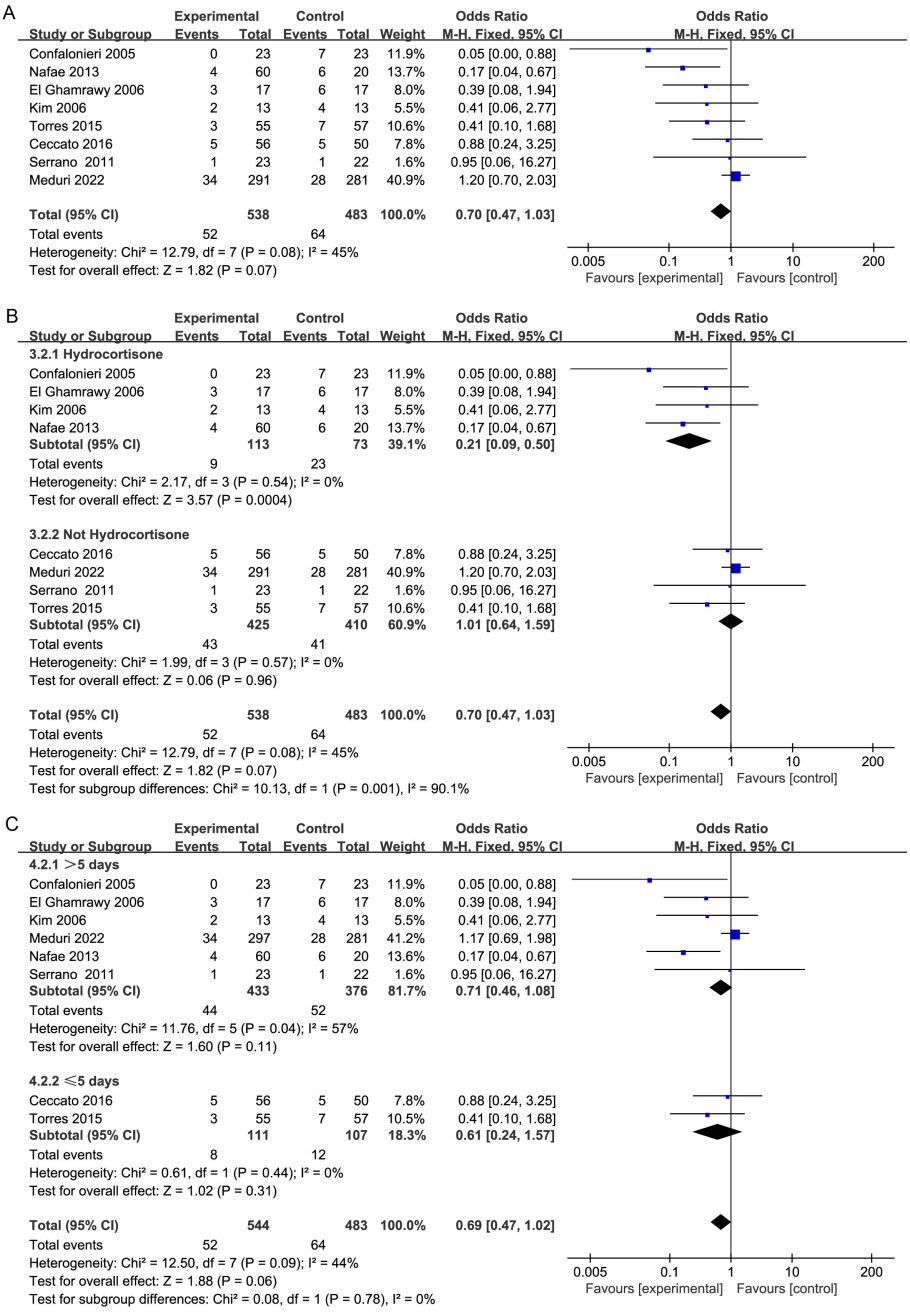
Meta-analysis comparing the risk of gastrointestinal hemorrhage **(A)**, frequency of hyperglycemia requiring treatment **(B)**, and incidence of hospital-acquired infection **(C)** between the study group treated with corticosteroids and the control group.

Eight studies reported hospital mortality (20, 29, 30, 32–36), and there was no apparent difference in mortality rates in hospitalization between both groups (OR, 0.70; 95% CI 0.47–1.03; *p* = 0.07, 8 studies, 1,021 patients, moderate certainty; Table 2, Figure 3A). The TSA-adjusted CIs were between 0.30 and 1.69, which is in agreement with the findings of the earlier study (Supplementary Figure S3). Cumulative Z-curves did not cross the conventional boundary values, nor did they cross the benefit boundary. Furthermore, there was no statistically significant difference in the effectiveness between the control group and the intervention group since the accumulated information gathered did not reach the anticipated level of RIS, and more RCTs are needed to prove this.

However, subgroup analysis revealed that patients treated with hydrocortisone (OR, 0.21; 95% CI 0.09–0.50; *p* = 0.0004; Figure 3B) may benefit more than those not treated with hydrocortisone (OR, 1.01; 95% CI 0.64–1.59; *p* = 0.96; Figure 3B). Furthermore, TSA indicated that further studies are required to confirm this finding (Supplementary Figures S4, S5).

Moreover, the duration of corticosteroid treatment was not linked to a noteworthy decrease in hospital mortality (Figure 3C).

#### ICU stay

**Figure 4:**
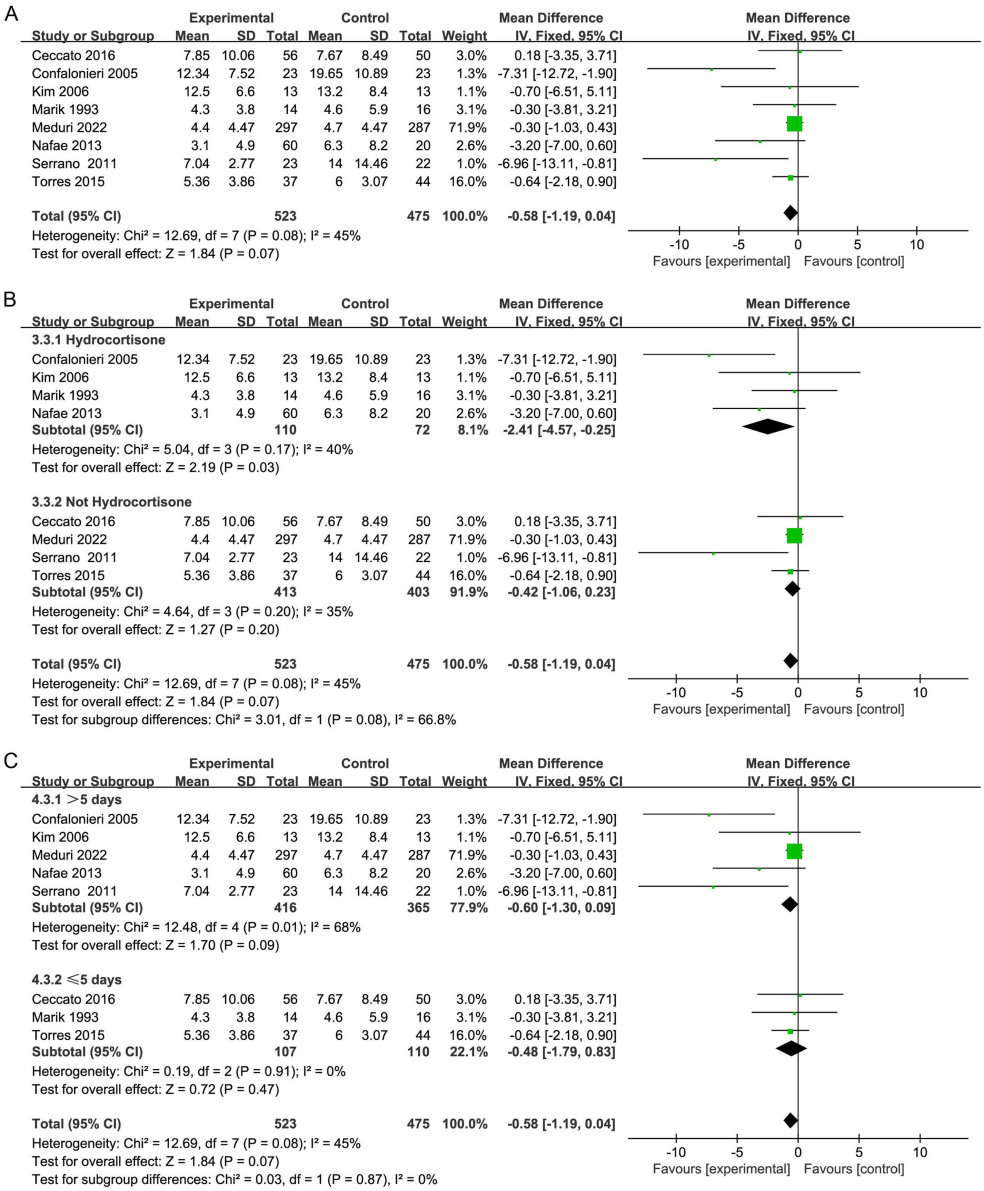
Meta-analysis of the length of MV time in patients receiving corticosteroid therapy versus the control group **(A)**, subgroup analysis of corticosteroid type **(B)**, and subgroup analysis of duration **(C)**.

The duration of ICU stay was reported in eight research (20, 29, 30, 32, 33, 35–37). There was no discernible variation in the duration of ICU stay between the control and intervention groups (MD, −0.58; 95% CI −1.19 to 0.04; *p* = 0.07; moderate certainty; Table 2; Figure 4A). Moreover, the TSA results were in agreement with the study results (TSA-adjusted CI, −3.09 to 1.93; Supplementary Figure S6). Cumulative Z-curves did not cross either the O’Brien-Fleming monitoring line or the conventional boundary. Furthermore, the cumulative information did not meet the futility thresholds or anticipated RIS. Consequently, there was no marked difference between the Control and intervention groups in terms of efficacy, and more RCTs are needed.

Furthermore, subgroup analysis presented that the application of hydrocortisone (OR, −2.41; 95% CI −4.57 to −0.25; *p* = 0.03; Figure 4B) significantly improved the length of ICU stay in contrast to that in the control group (Supplementary Figure S7), and the nonhydrocortisone treatment group did not show this finding (OR, −0.42; 95% CI −1.06 to 0.23; *p* = 0.20; Figure 4B; Supplementary Figure S8). Subgroup analysis did not reveal that the duration of corticosteroid treatment affected the ICU length of stay (Figure 4C).

#### Hospital stay

**Figure 5:**
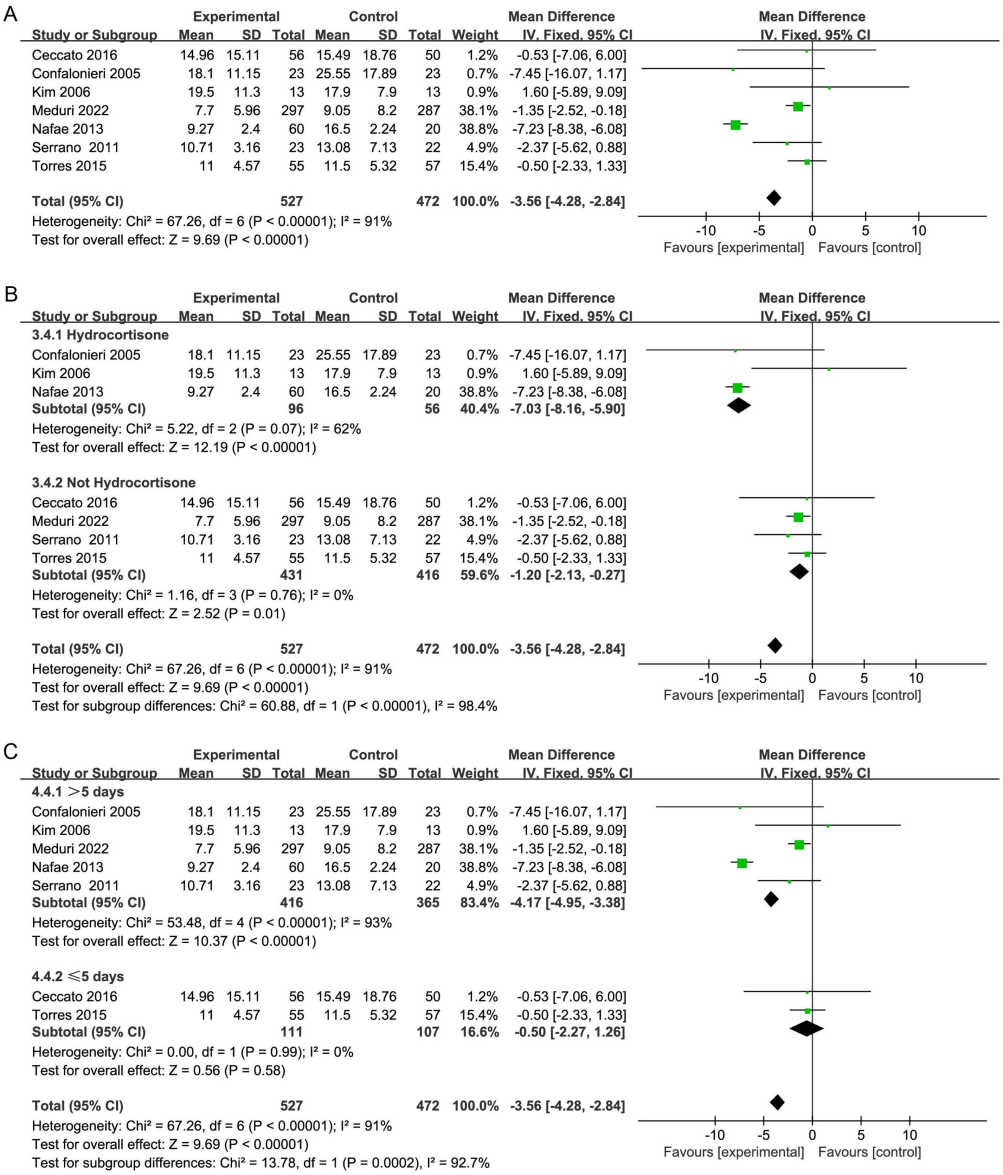
Meta-analysis of the need for MV in patients receiving corticosteroid therapy versus the control group **(A)**, subgroup analysis of corticosteroid type **(B)**, and subgroup analysis of duration **(C)**.

Seven studies (20, 29, 30, 32, 33, 35, 36) demonstrated that hospitalization was shorter in the corticosteroid group in contrast to the control group (MD, −3.56; 95% CI −4.28 to −2.84; *p* < 0.01; low certainty; Table 2; Figure 5A). TSA identified that the RIS was not met; nevertheless, the Z-curve crossed the benefit boundaries, indicating that corticosteroids were more beneficial than the control treatment (Supplementary Figure S9).

Furthermore, subgroup analysis revealed that both hydrocortisone and nonhydrocortisone reduced the length of hospital stay (Figure 5B; Supplementary Figures S10, S11). A duration of corticosteroid therapy longer than 5 days may reduce hospital stay (MD, −4.17; 95% CI −4.95 to −3.38; *p* < 0.001; Figure 5C).

#### Need for MV and MV time

**Figure 6:**
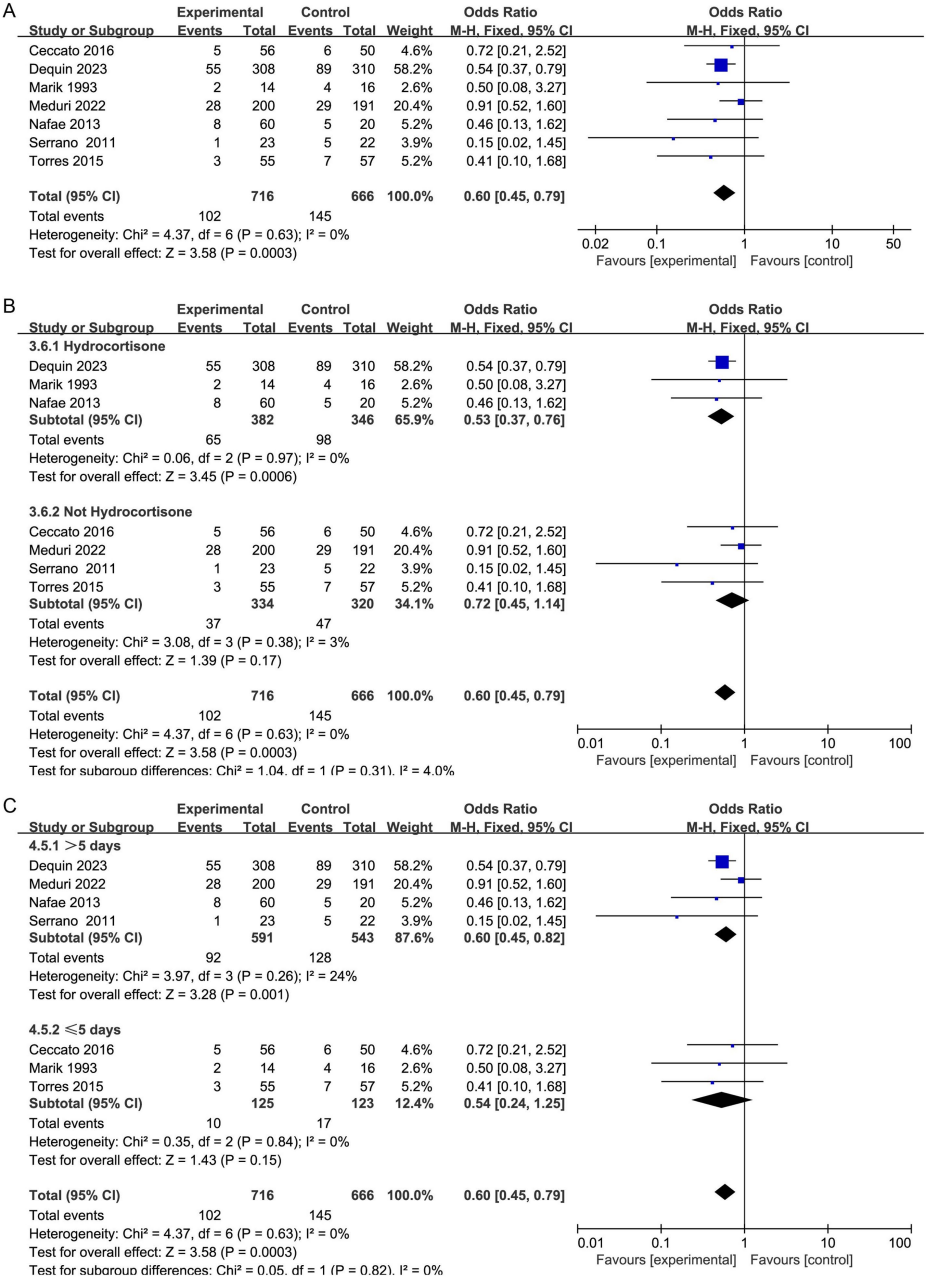
Meta-analysis of the CRP level in patients receiving corticosteroid therapy versus the control group **(A)**, subgroup analysis of corticosteroid type **(B)**, and subgroup analysis of duration **(C)**.

**Figure 7:**
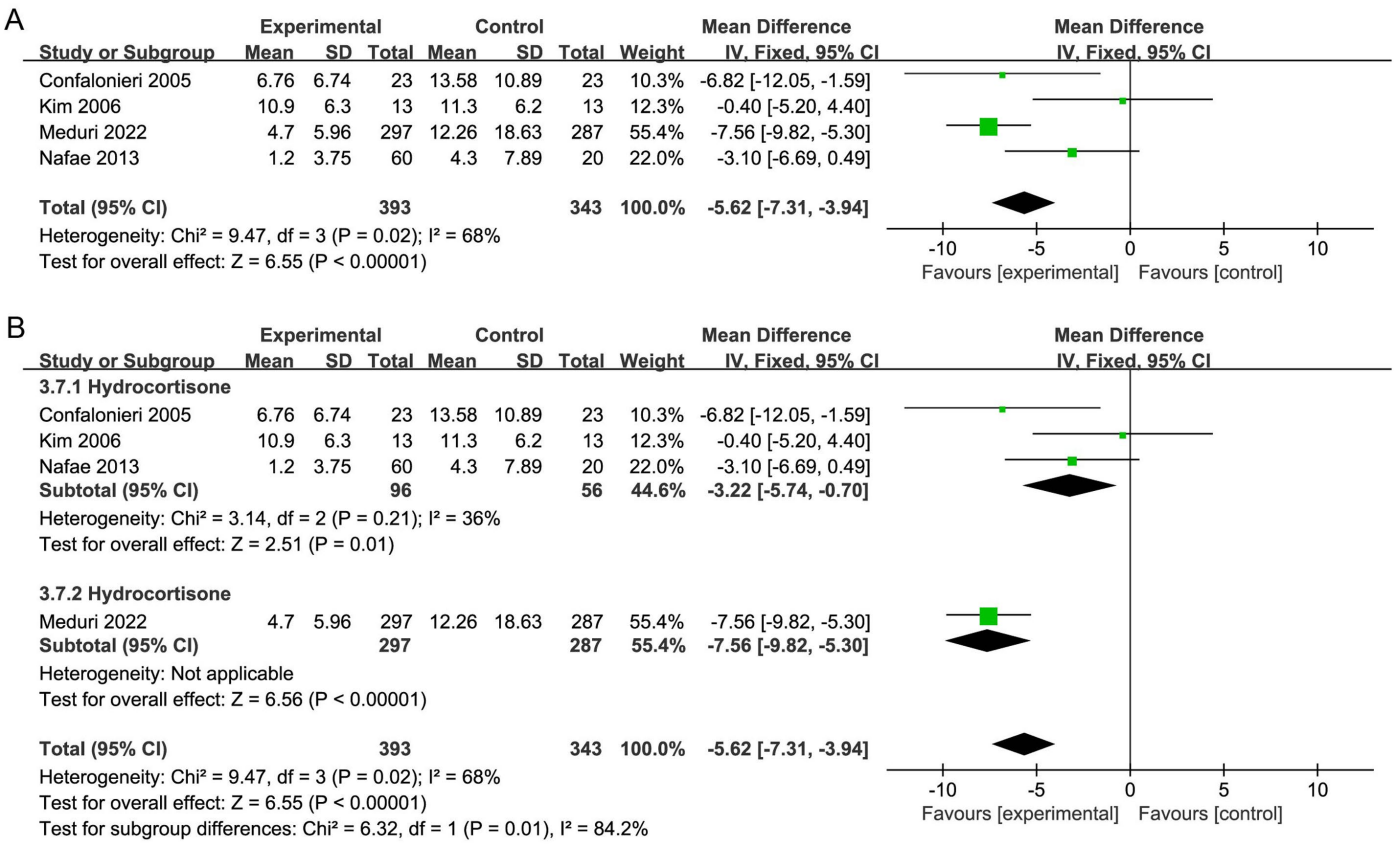
Meta-analysis of short-term mortality in individuals taking corticosteroid treatment versus control group **(A)**, subgroup analysis of corticosteroid type **(B)**.

Seven studies reported the need for MV (19, 20, 29, 30, 32, 33, 37), and four studies reported the duration of MV (20, 32, 35, 36). Corticosteroid therapy was linked to a decrease in the need for MV (OR, 0.60; 95% CI 0.45–0.79; *p* = 0.0003; moderate certainty; Table 2; Figure 6A) and MV duration (MD, −5.62; 95% CI −7.31 to −3.94; *p* < 0.001; moderate certainty; Table 2; Figure 7A). The TSA-adjusted CIs were 0.41–0.88 for the need for MV (Supplementary Figure S12), and the Z-curve crossed the benefit boundaries, indicating that corticosteroid treatment was more beneficial than the control treatment. The TSA-adjusted CIs ranged from −9.1 to −2.2 with regard to the duration of MV, and the Z-curve crossed the RIS and the conventional boundary, indicating that corticosteroids were more beneficial than the control treatments (Supplementary Figure S13).

Subgroup analysis with regard to the type of corticosteroids showed that hydrocortisone was more beneficial for the need for MV (OR, 0.53; 95% CI 0.37–0.76; *p* = 0.0006; Figure 6B; Supplementary Figure S14). Moreover, hydrocortisone subgroup was linked to shorter MV duration (MD, −3.22; 95% CI −5.74 to −0.70; *p* = 0.01; Figure 7B; Supplementary Figure S15), but the nonhydrocortisone subgroup did not show improvement (Supplementary Figure S16).

Furthermore, subgroup analysis of the duration of corticosteroid therapy showed that corticosteroid therapy longer than 5 days may reduce the need for MV (OR, 0.60; 95% CI 0.45–0.82; *p* = 0.001; Figure 6C).

#### CRP level

**Figure 8:**
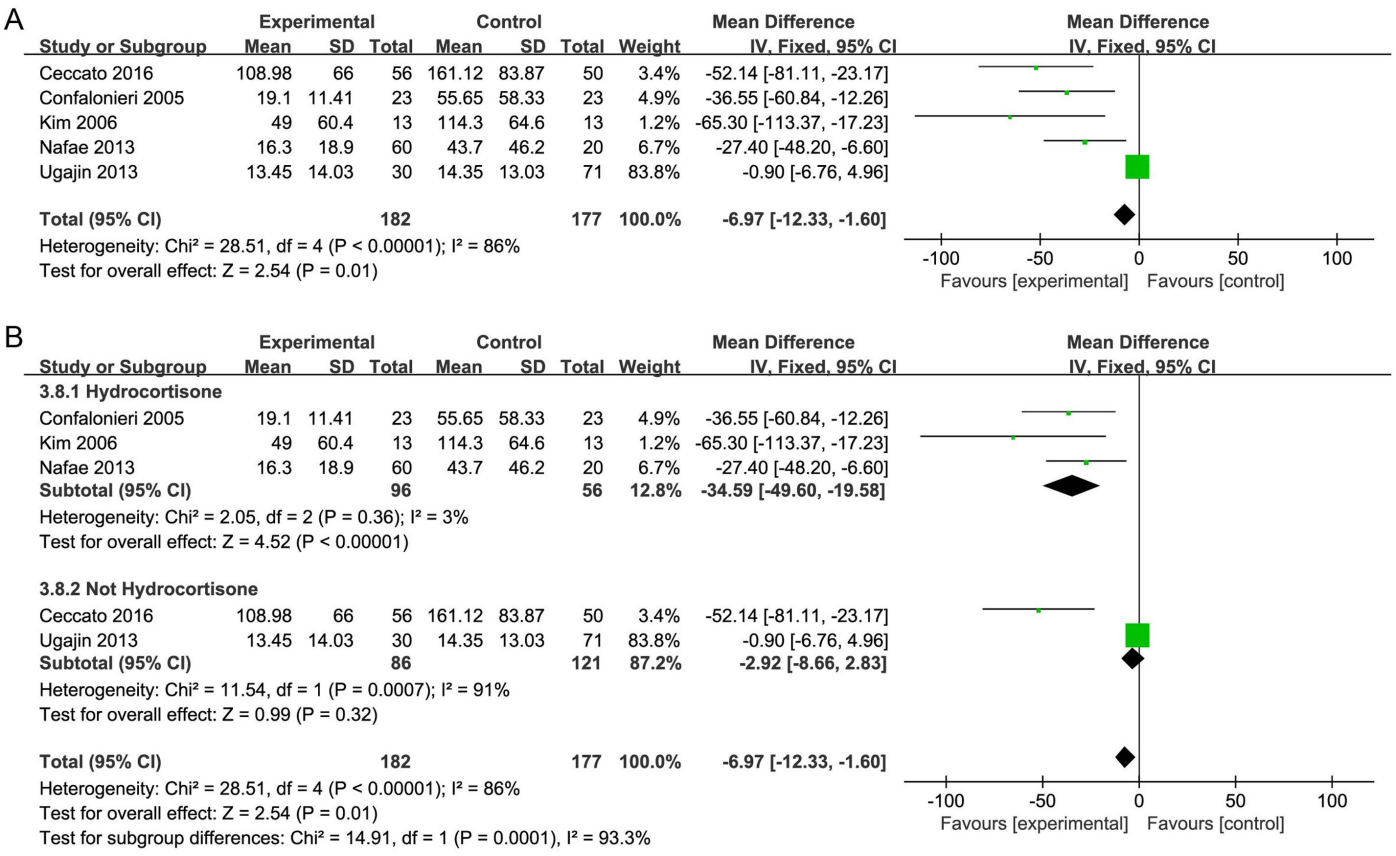
Meta-analysis of in-hospital mortality in individuals taking corticosteroid treatment versus control group **(A)**, subgroup analysis of corticosteroid type **(B)**.

Five studies (29, 31, 32, 35, 36) reported the CRP level, and the results showed that CRP level declined more in the corticosteroid group versus the control group (MD, −6.97; 95% CI −12.33 to −1.60; *p* < 0.05; very low certainty; Table 2; Figure 8A). The TSA-adjusted CIs ranged from −20.4 to 6.5 (Supplementary Figure S17). The cumulative Z-curve did not reach the O’Brien-Fleming monitoring line for benefit, although it did pass the traditional line. Furthermore, neither the RIS nor the futility were crossed by the cumulative Z-curve, suggesting that there may have been false-positive results. Therefore, further large RCTs are needed to prove this hypothesis.

Furthermore, subgroup analysis with regard to the type of corticosteroids exhibited that hydrocortisone treatment caused a considerable decrease in the level of CRP (MD, −34.59; 95% CI −49.60 to −19.58; *p* <0.001; Figure 8B), and the TSA results were in agreement (Supplementary Figure S18).

#### Severe adverse events

**Figure 9:**
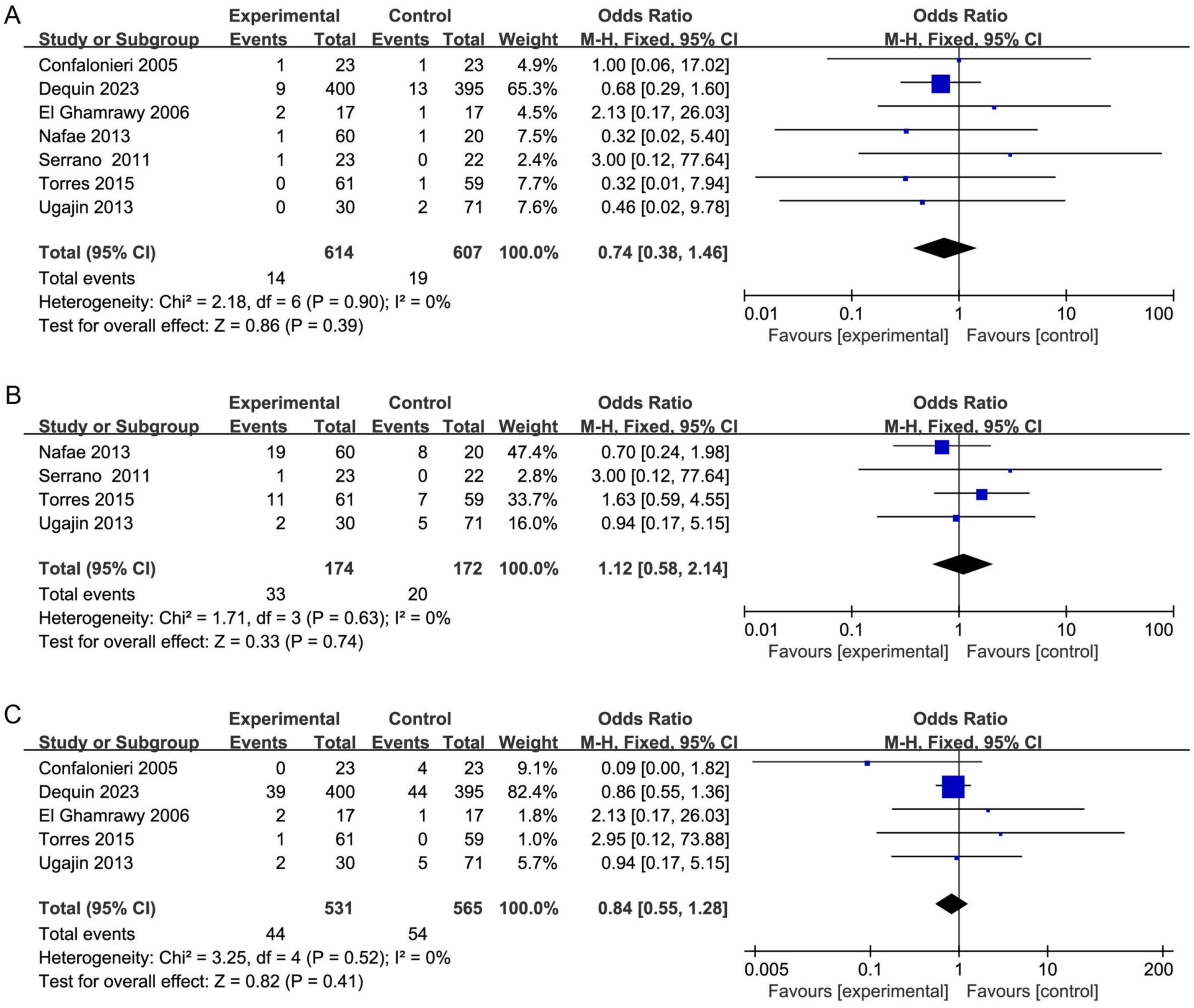
Meta-analysis of duration of stay in ICU in individuals taking corticosteroid treatment versus control group **(A)**, subgroup analysis of corticosteroid type **(B)**, and subgroup analysis of duration **(C)**.

Among the included researches, no significant differences with regard to severe adverse effects were found between the two groups (Figure 9). There were similar risks of gastrointestinal hemorrhage (7 studies, 1,221 patients; OR, 0.74; 95% CI 0.38 to 1.46; *p* = 0.39; I^2^ = 0%), frequency of hyperglycemia requiring treatment (4 studies, 346 patients; OR, 1.12; 95% CI 0.58 to 2.14; *p* = 0.74; I^2^ = 0%), and incidence of hospital-acquired infection (5 studies, 1,096 patients; OR, 0.84; 95% CI 0.55 to 1.28; *p* = 0.41; I^2^ = 0%).

## Discussion

It is unknown if adjunctive corticosteroid therapy is safe and effective in treating individuals with sCAP. In fact, there seems to be a survival advantage linked to early corticosteroid therapy in patients with ARDS, coronavirus illness 2019, and septic shock (5, 38, 39). The underlying pathophysiology, driven by lung inflammation, is similar among sCAP and the above diseases. Hence, it is reasonable to extrapolate their potential benefits to patients with CAP.

Recently, the impact of corticosteroid therapy for sCAP was examined in two sizable RCTs, however the findings were debatable (19, 20). The CAPE COD trial showed that hydrocortisone could be expected to reduce mortality in sCAP individuals (RR 0.53 [95% CI 0.33–0.84]) (19). The ESCAPe trial demonstrated that methylprednisolone has no apparent benefit in sCAP individuals (adjusted OR 0.90, 95% CI 0.57–1.40) (20). It was noted that systemic corticosteroids were linked to improved clinical results, especially treatment with hydrocortisone, according to subgroup analyses. This is the most recent meta-analysis on the topic that we are aware of, and we identified that sCAP patients undergoing hydrocortisone treatment had markedly better outcomes than those who did not receive hydrocortisone treatment.

In this up-to-date meta-analysis, we included 1959 patients from 11 studies who fulfilled the predefined criteria. In our included studies, seven studies (20, 31, 32, 34–37) did not report the specific pathogens. Three trials (19, 29, 30) ruled out influenza infection and one study (33) excluded active mycobacterial or fungal infection. As few included RCTs reported the specific pathogen, a further subgroup meta-analysis with regards to the type of pneumonia (bacterial, viral, influenza) could not be performed. Overall, our meta-analysis revealed that in contrast to the individuals in the control group, patients in corticosteroids group had considerably decreased short-term mortality and CRP levels. TSA demonstrated the cumulative Z-curve crossed the traditional benefits monitoring line, but not the O’Brien-Fleming monitoring line. This suggests that there may be false-positive results in the findings, and further large scale RCTs are needed to verify the authenticity of the results.

In order to avoid the influence of other confounding factors, we conducted a subgroup analysis. Moreover, we observed that corticosteroids could shorter hospital stays, reduce MV demand, and MV duration. On the other hand, the duration of hospital stay in the ICU did not considerably decrease. In addition, we did not find that the use of corticosteroids increased the incidence of adverse events. Subgroup analysis revealed that all of the above studies benefited from hydrocortisone treatment. The TSA results were consistent with regard to short-term mortality, length of hospital stay, duration of MV and CRP level. Moreover, it is reassuring that corticosteroid therapy did not raise the prevalence of adverse effects. Therefore, based on our study, hydrocortisone should be chosen when administering hormone therapy to patients with sCAP, while the use of non-hydrocortisone treatment for specific populations requires further study.

In addition, the subgroup analysis results of a recently published meta-analysis (40) revealed that hydrocortisone treatment resulted in a considerable decrease in all-cause mortality (OR, 0.48; 95% CI 0.30–0.72), but no benefits were shown for methylprednisolone (OR, 0.79; 95% CI 0.57–1.08). The authors did not focus much on this subgroup result and conducted only one mortality analysis, not including the results of two recent large-scale RCTs (19, 20). In our study, we conducted a comprehensive subgroup analysis, and the findings indicated that for sCAP individuals, the mortality in hydrocortisone group was much lower than the non-hydrocortisone group.

However, another recent meta-analysis came to a different conclusion (41). According to Saleem et al., there is no discernible mortality difference between individuals on corticosteroid medication and those on standard care (relative risk, 0.85; 95% CI 0.67–1.07, *p* = 0.17). It is possible that variations in the criteria for population inclusion account for the discrepancy between our research and the earlier meta-analysis, which included both non-sCAP and sCAP patients in their study.

Pitre et al. implemented a meta-analysis focusing on corticosteroids via pairwise and dose–response analyses (42). They discovered that corticosteroids decreased patients’ deaths who had severe pneumonia and decreased the need for invasive MV and ICU admission. The above conclusions are consistent with our observations in sCAP patients, but they focused on bacterial community-acquired pneumonia.

In pharmacological, hydrocortisone functions as a glucocorticoid with both mineralocorticoid and glucocorticoid effects, whereas dexamethasone and methylprednisolone act as synthetic glucocorticoids primarily exerting glucocorticoid effects, with minimal mineralocorticoid activities (43). In summary, hydrocortisone may improve cardiovascular function by restoring effective blood volume through increased mineralocorticoid activity and regulating homeostasis to balance sodium and potassium (44, 45). The dual effect of hydrocortisone, encompassing both glucocorticoid and mineralocorticoid actions, may offer distinct therapeutic benefits, especially in the context of sCAP. Additionally, hydrocortisone is a low-potency and short-acting glucocorticoid, while prednisolone, and methylprednisolone are long-acting corticosteroids that exhibit higher potency than hydrocortisone (43). Hydrocortisone regulates the immune response and reducing inflammation without inducing excessive immune dysregulation, unlike other corticosteroids that can lead to prolonged immunosuppression (46). Therefore, these characteristics of hydrocortisone may contribute to the observed mortality benefits in sCAP (47).

In conclusion, our meta-analysis included the largest sample size according to current international sCAP inclusion standards. In light of the disparate results of two recent RCTs, we performed subgroup analyses in accordance with corticosteroid type. From the perspective of pathophysiology, although different types of corticosteroids are dose-equivalent, the choice of corticosteroids can vary due to differences in terms of efficacy and pharmacokinetic characteristics. In fact, these corticosteroids are not exactly the same in terms of efficacy and pharmacokinetic characteristics. Our meta-analysis demonstrated the benefits of hydrocortisone treatment compared with other types of corticosteroids. This discovery has significant implications for informing the design of RCTs.

With regard to treatment duration, we implemented a subgroup analysis based on the most recent sCAP management guidelines (1). However, there is no unified standard for the dosage and start time of corticosteroid use, and further research is needed. As sCAP is a clinically common syndrome with high heterogeneity, and the patients included in the present study represented a heterogeneous population. Furthermore, treatment protocols and guideline recommendations varied among studies. Hence, there are great challenges in standardizing treatment regimens of corticosteroid use. In our meta-analysis, we found that in terms of reduced need for MV and shorter hospital stays, a treatment duration of more than 5 days is more beneficial than a duration of less than or equal to 5 days. Further RCT studies with regards to the standard dosing and timing of corticosteroids in sCAP are needed.

Though our meta-analysis showed that corticosteroids might be clinical beneficial for sCAP, there were still some aspects unresolved. Firstly, based on our analysis, future RCTs investigating which type of corticosteroids is more effective in treating sCAP are expected. Secondly, future RCTs investigating the effect of corticosteroids in sCAP with different types of pathogens (bacterial, viral, influenza) are meaningful. Thirdly, further studies exploring biomarkers that could assist in personalized treatment of corticosteroids in sCAP are clinical useful.

Our meta-analysis has several strengths. Firstly, only sCAP studies were included in our studies, which avoided the confounding effects, such as severe hospital acquired pneumonia and disease severity. Secondly, 11 studies were included in our meta-analysis, as our known, this is the largest sample size so far. Thirdly, TSA analyses were used to robust our results.

There exist some limitations to this meta-analysis. First of all, the patients in this research represented a heterogeneous sample because sCAP is a clinically frequent condition with considerable heterogeneity. For instance, some studies used the ATS standard, other studies used the BTS standard, some studies included patients with immune deficiencies, and other studies included patients with COVID-19 (19, 20, 29–37). Second, there is no unified standard for the start time of corticosteroid use, and we lacked data to perform subgroup analysis with regard to this aspect. Third, the sample sizes of the studies included were small, and those included were predominantly single-center trials, which may have produced bias.

## Conclusion

Our meta-analysis demonstrated that corticosteroids, especially hydrocortisone, can reduce the mortality of sCAP patients.

## Data Availability

The original contributions presented in the study are included in the article/Supplementary material, further inquiries can be directed to the corresponding authors.
