# Supplementary material for: The effect and safety of corticosteroid treatment for severe community-acquired pneumonia: a meta-analysis of randomized controlled trials
Source: Front Med (Lausanne). 2024 Nov 6;11:1457469. doi: 10.3389/fmed.2024.1457469 (PMC11576290; doi:10.3389/fmed.2024.1457469)
Supplement: Supplementary file 1 [file Data_Sheet_1.docx]

**Supplementary Material: Searching strategies and subgroup analyses**

**Search strategy**

**Pubmed (result 74)**

((dexamethasone[Title/Abstract]) OR ((((((((((((("Adrenal Cortex Hormones"[Mesh]) OR (Hormones, Adrenal Cortex[Title/Abstract])) OR (Corticosteroids[Title/Abstract])) OR (Corticosteroid[Title/Abstract])) OR (Corticoids[Title/Abstract])) OR (Corticoid[Title/Abstract])) OR (Adrenal Cortex Hormone[Title/Abstract])) OR (Cortex Hormone, Adrenal[Title/Abstract])) OR (Hormone, Adrenal Cortex[Title/Abstract])) OR (Prednisone[Title/Abstract])) OR (Hydrocortisone[Title/Abstract])) OR (Prednisolone[Title/Abstract])))) AND (Severe Community Acquired Pneumonia[Title/Abstract])

**Web of science （result 298）**

TS=(Adrenal Cortex Hormones OR Hormones, Adrenal Cortex OR Corticosteroids OR Corticosteroid OR Corticoids OR Corticoid OR Adrenal Cortex Hormone OR Cortex Hormone, Adrenal OR Hormone, Adrenal Cortex OR Prednisone OR Hydrocortisone OR Prednisolone OR dexamethasone)

TS=(Severe Community Acquired Pneumonia)

**Cochrane（result 59）**

(Adrenal Cortex Hormones):ti,ab,kw OR (Hormones, Adrenal Cortex):ti,ab,kw OR (Corticosteroids):ti,ab,kw OR (Corticosteroid):ti,ab,kw OR (Corticoids):ti,ab,kw OR (Corticoid):ti,ab,kw OR (Adrenal Cortex Hormone):ti,ab,kw OR (Cortex Hormone, Adrenal):ti,ab,kw OR (Hormone, Adrenal Cortex):ti,ab,kw OR (Prednisone):ti,ab,kw OR (Hydrocortisone):ti,ab,kw OR (Prednisolone):ti,ab,kw OR (dexamethasone):ti,ab,kw

(Severe Community Acquired Pneumonia):ti,ab,kw

**EMBASE（result 95）**

‘Adrenal Cortex Hormones’:ab,ti OR ‘Hormones, Adrenal Cortex’:ab,ti OR ‘Corticosteroids’:ab,ti OR ‘Corticosteroid’:ab,ti OR ‘Corticoids’:ab,ti OR ‘Corticoid’:ab,ti OR ‘Adrenal Cortex Hormone’:ab,ti OR ‘Cortex Hormone, Adrenal’:ab,ti OR ‘Hormone, Adrenal Cortex’:ab,ti OR ‘Prednisone’:ab,ti OR ‘Hydrocortisone’:ab,ti OR ‘Prednisolone’:ab,ti OR ‘dexamethasone’:ab,ti AND Severe Community Acquired Pneumonia:ab,ti


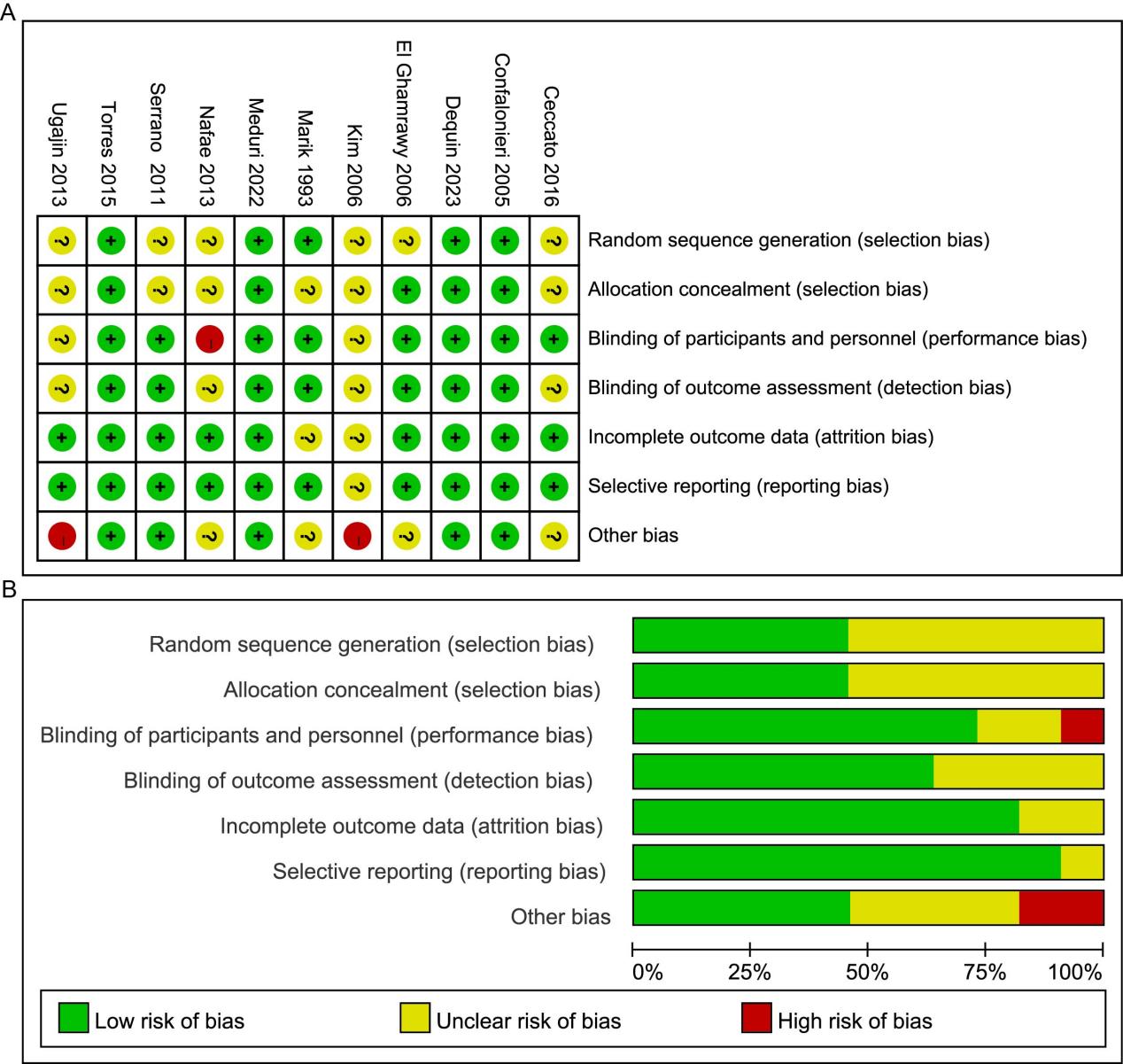


**Figure S1. Risk of bias assessment.**


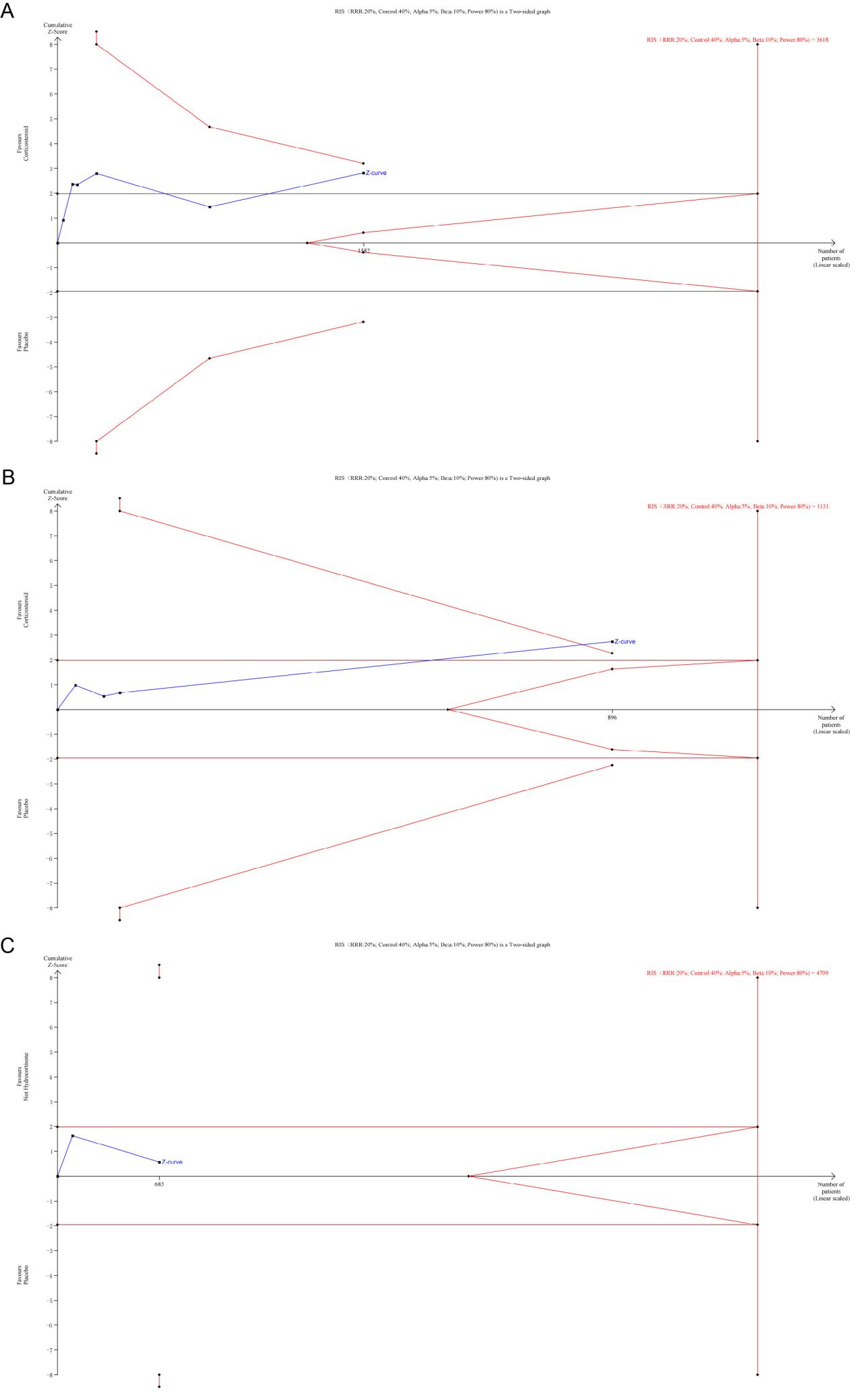


**Figure S2. Trial sequential analysis for short-term mortality.** All included trials receiving corticosteroids therapy versus control group (A), subgroup analysis of corticosteroids type (B), subgroup analysis of duration time (C).

The blue line represents the cumulative Z-curve. The red line is the O’Brian-Fleming monitoring line (TSA) and the futility line. The purple lines are conventional P = .05 lines. The required sample size for a conclusive result was 3618(A), 1131(B), 4709(C) .

1. Z-curve crossed conventional P = .05 lines , but did not reach O’Brian-Fleming monitoring line indicates that available results support corticosteroids therapy more beneficial than the control group results but need further studies to avoid false positive results.
2. Z-curve crossing over the O’Brian-Fleming monitoring boundaries indicates that hydrocortisone therapy is more beneficial than the control group in severe community-acquired pneumonia patients.

C.Z-curve did not crossed conventional P = .05 lines indicates that there was no statistically significant difference in efficacy between the non-hydrocortisone therapy and the control group.


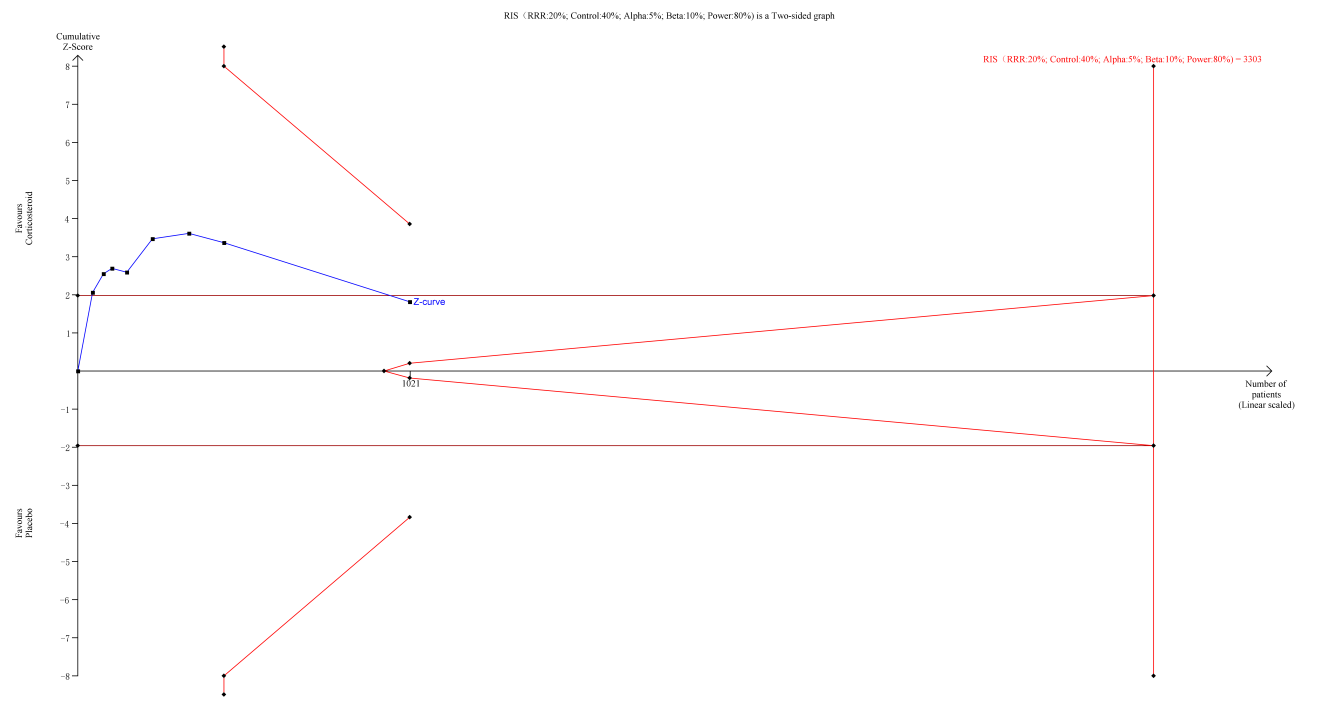


**Figure S3.Trial sequential analysis for In-hospital mortality in included trials receiving corticosteroids therapy versus control group.**

The blue line represents the cumulative Z-curve. The red line is the O’Brian-Fleming monitoring line (TSA) and the futility line. The purple lines are conventional P = .05 lines. The required sample size for a conclusive result was 3303. Z-curve does not cross the P = .05 lines, nor does it cross the O’Brian-Fleming monitoring line and did not reach the RIS. There was no statistically significant difference in efficacy between the corticosteroids therapy and the control group.

**
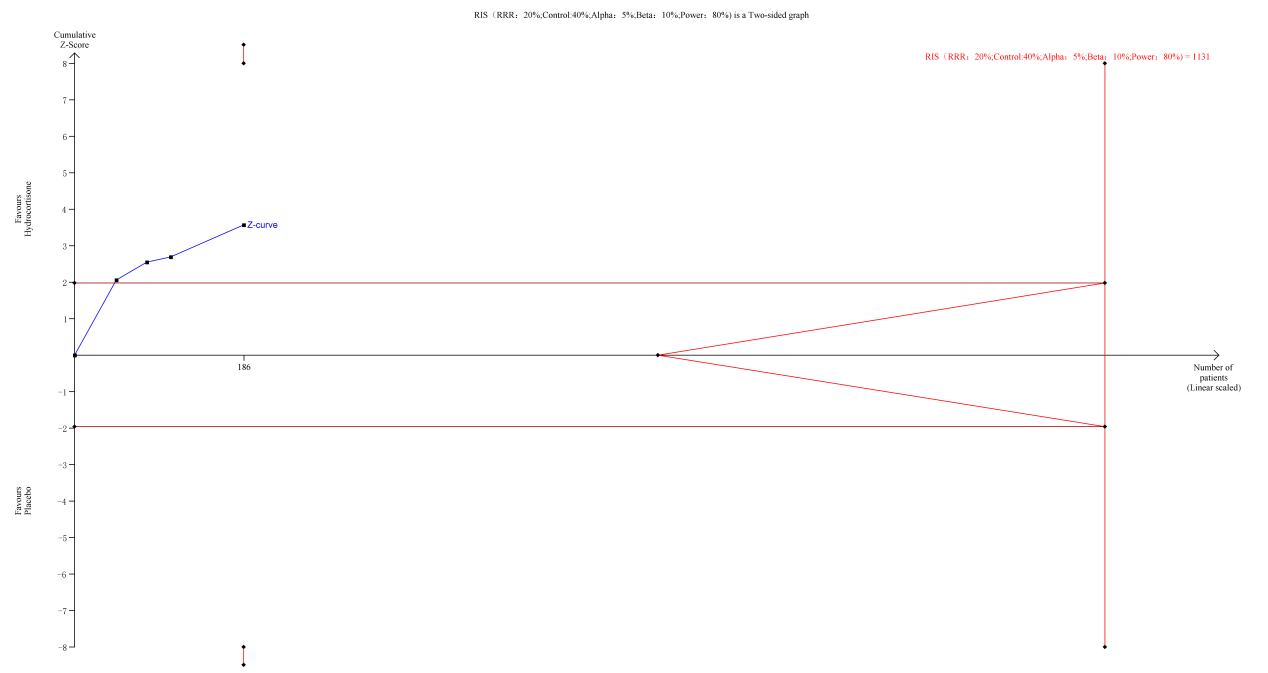
**

**Figure S4.Trial sequential analysis for In-hospital mortality in included trials receiving hydrocortisone therapy versus control group.** The blue line represents the cumulative Z-curve. The red line is the O’Brian-Fleming monitoring line (TSA) and the futility line. The purple lines are conventional P = .05 lines. The required sample size for a conclusive result was 1131. Z-curve crossed conventional P = .05 lines, but did not reach O’Brian-Fleming monitoring line indicates that available results support hydrocortisone therapy more beneficial than the control group results but need further studies to avoid false positive results.

**
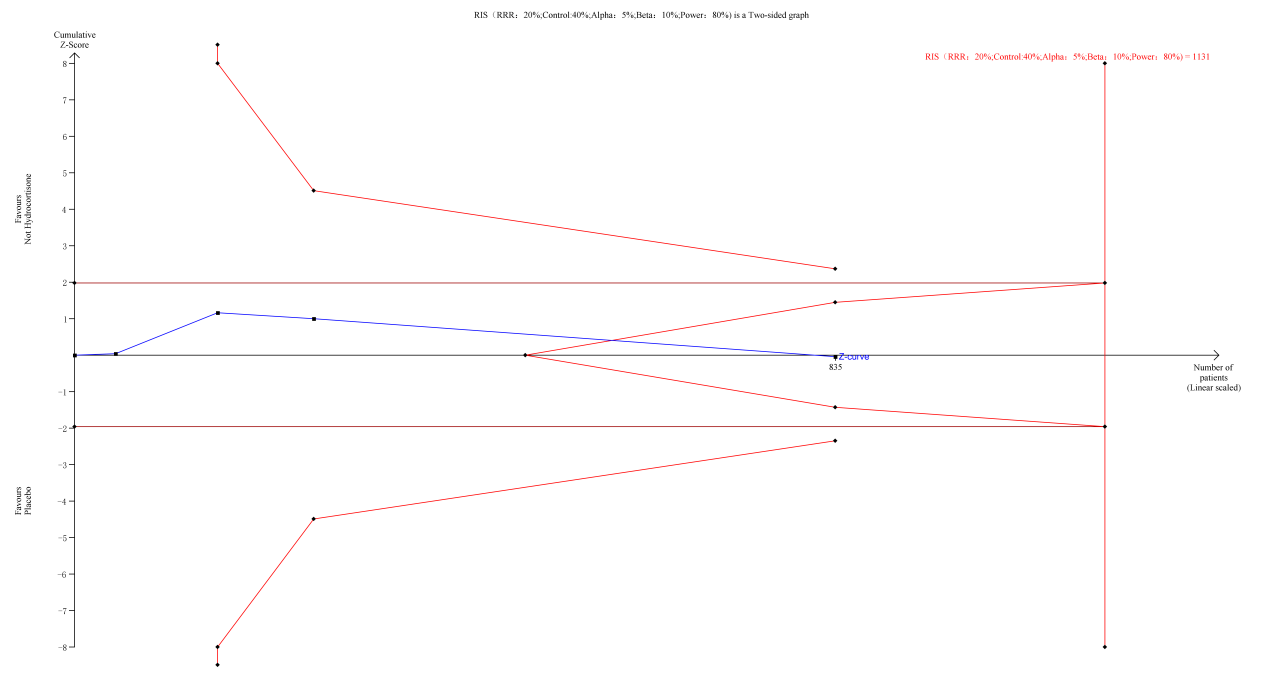
**

**Figure S5. Trial sequential analysis for In-hospital mortality in included trials receiving non-hydrocortisone therapy versus control group.** The blue line represents the cumulative Z-curve. The red line is the O’Brian-Fleming monitoring line (TSA) and the futility line. The purple lines are conventional P = .05 lines. The required sample size for a conclusive result was 1131. Z-curve does not cross the P = .05 lines, nor does it cross the O’Brian-Fleming monitoring line and did not reach the RIS. There was no statistically significant difference in efficacy between the non-hydrocortisone therapy and the control group.

**
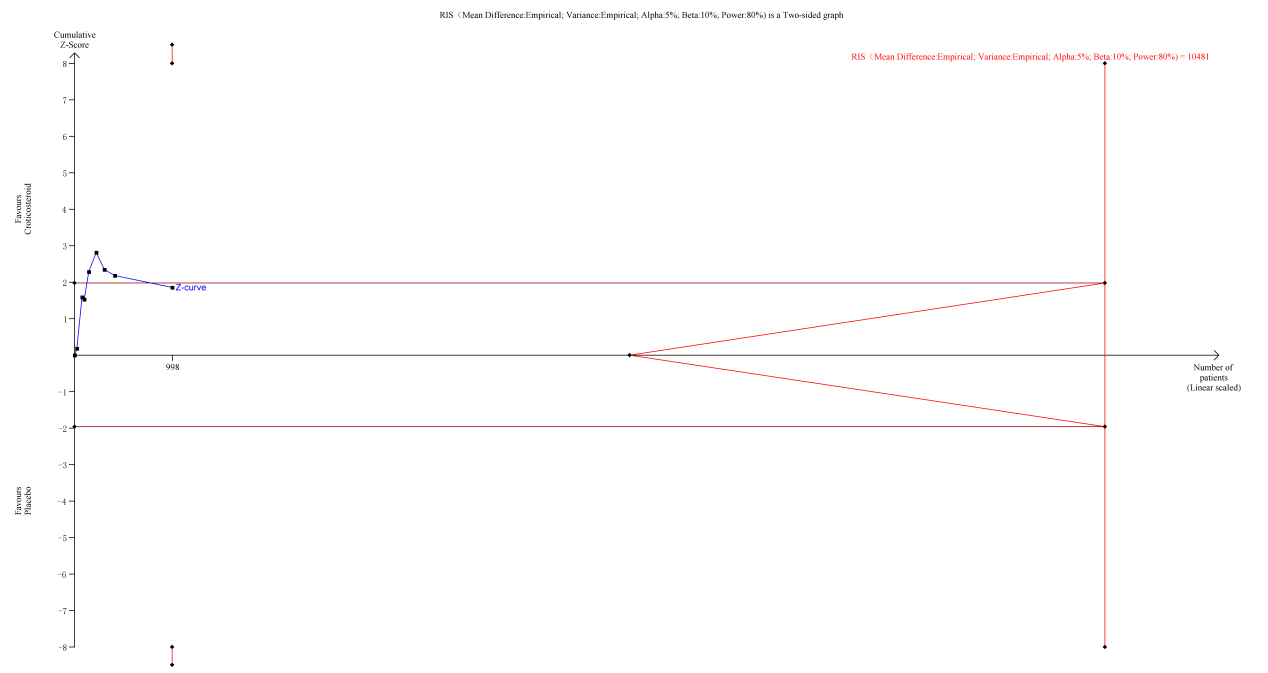
**

**Figure S6.Trial sequential analysis for the length of ICU stay in included trials receiving corticosteroids therapy versus control group.**The blue line represents the cumulative Z-curve. The red line is the O’Brian-Fleming monitoring line (TSA) and the futility line. The purple lines are conventional P = .05 lines. The required sample size for a conclusive result was 10481. Z-curve does not cross the P = .05 lines, nor does it cross the O’Brian-Fleming monitoring line and did not reach the RIS. There was no statistically significant difference in efficacy between the corticosteroids therapy and the control group and need for further studies to prove it.

**
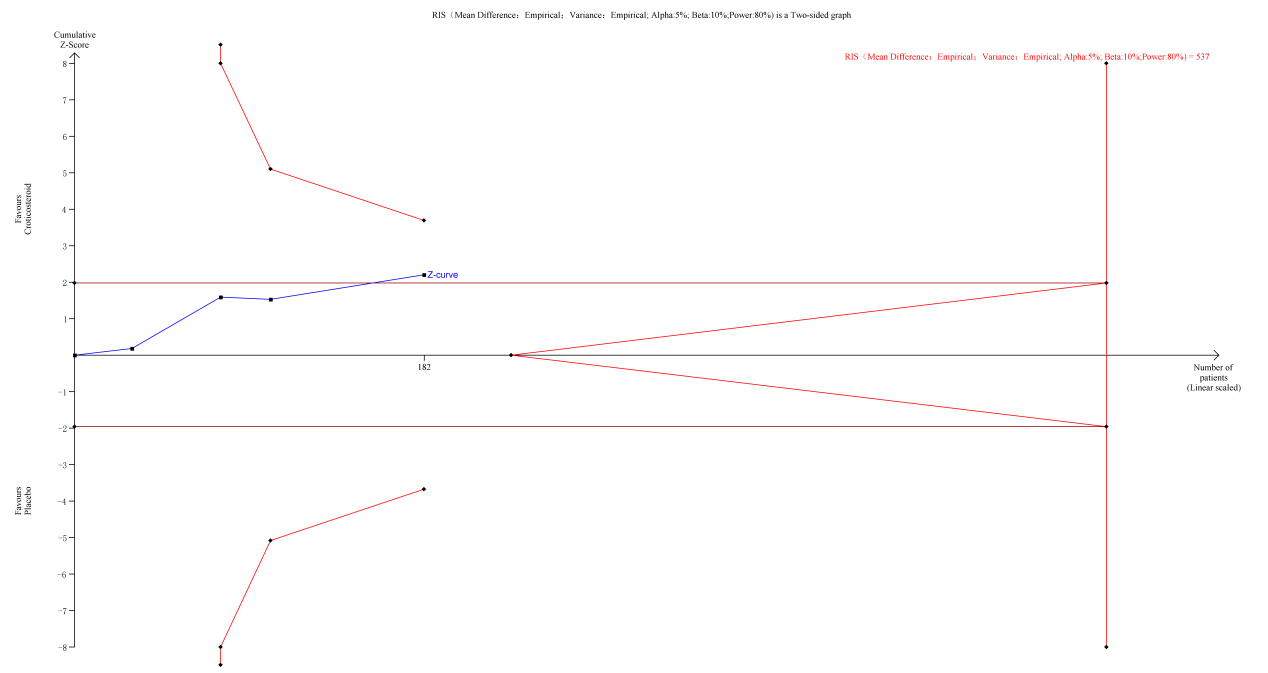
**

**Figure S7.Trial sequential analysis for the length of ICU stay in included trials receiving hydrocortisone therapy versus control group.** The blue line represents the cumulative Z-curve. The red line is the O’Brian-Fleming monitoring line (TSA) and the futility line. The purple lines are conventional P = .05 lines. The required sample size for a conclusive result was 537. Z-curve crossed conventional P = .05 lines , but did not reach O’Brian-Fleming monitoring line indicates that available results support hydrocortisone therapy more beneficial than the control group results but need further studies to avoid false positive results.


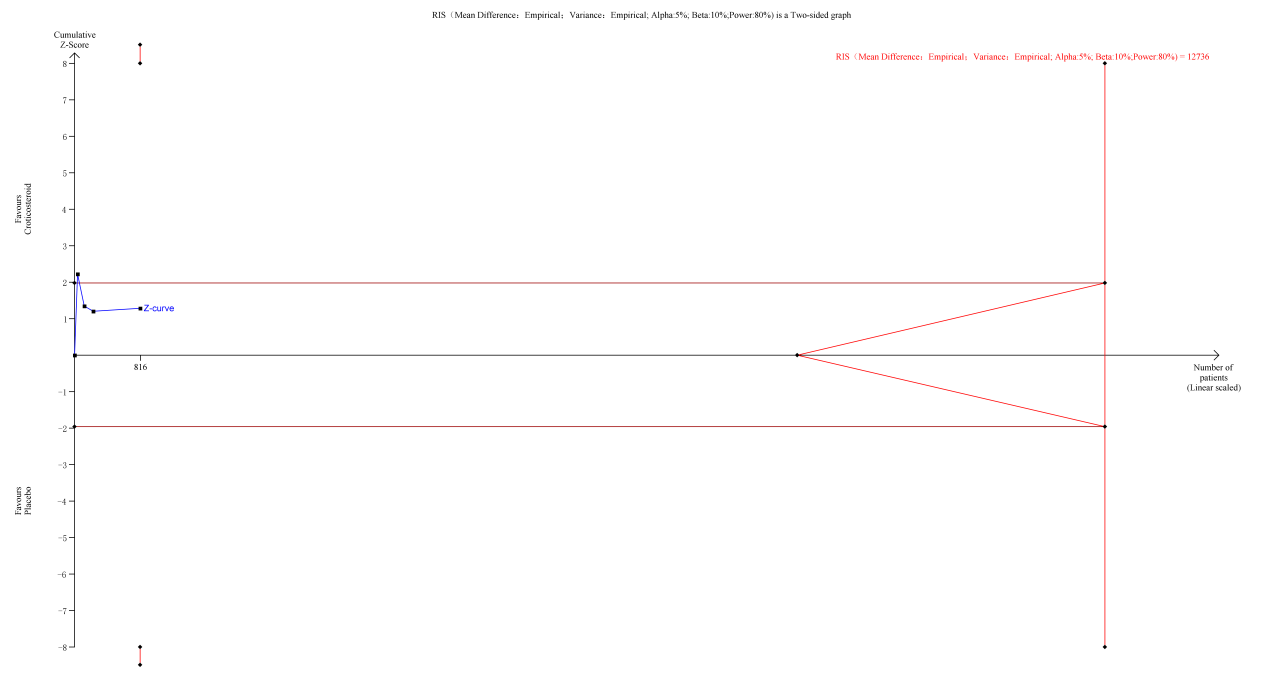
**Figure S8.Trial sequential analysis for the length of ICU stay in included trials receiving non-hydrocortisone therapy versus control group.** The blue line represents the cumulative Z-curve. The red line is the O’Brian-Fleming monitoring line (TSA) and the futility line. The purple lines are conventional P = .05 lines. The required sample size for a conclusive result was 12736. Z-curve does not cross the P = .05 lines, nor does it cross the O’Brian-Fleming monitoring line and did not reach the RIS. There was no statistically significant difference in efficacy between the non-corticosteroids therapy and the control group.

**
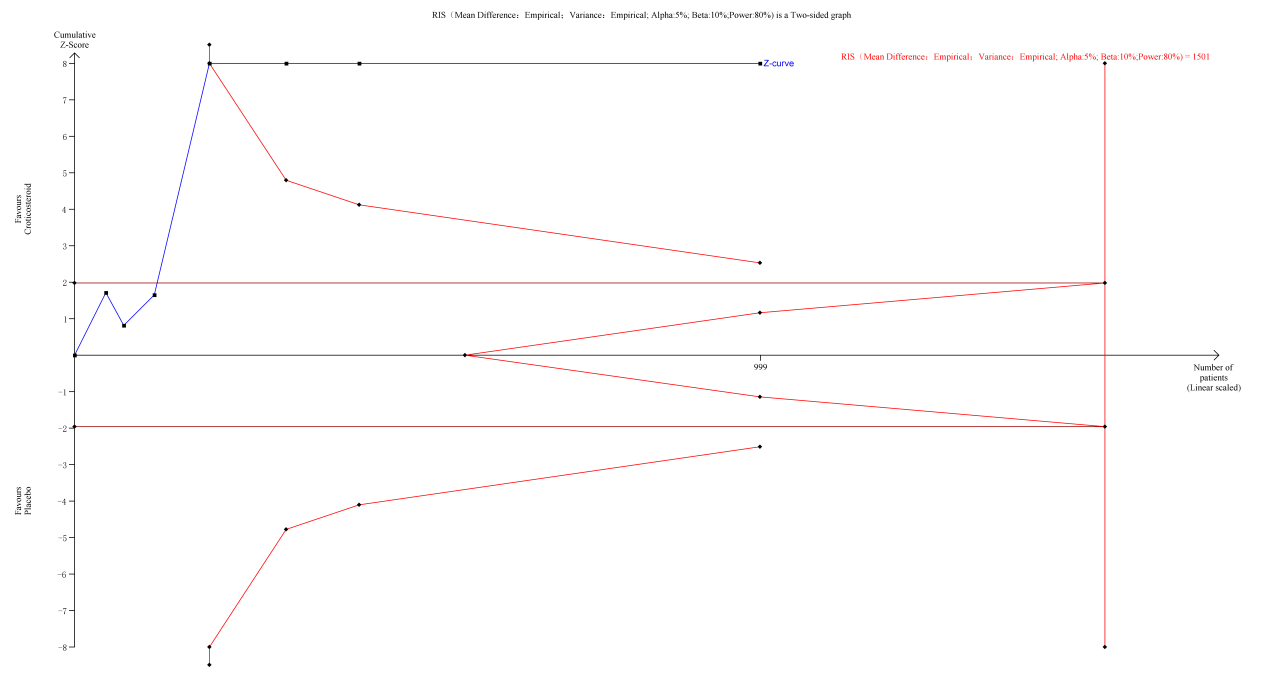
Figure S9.Trial sequential analysis for the length of hospital stay in included trials receiving corticosteroids therapy versus control group.**

The blue line represents the cumulative Z-curve. The red line is the O’Brian-Fleming monitoring line (TSA) and the futility line. The purple lines are conventional P = .05 lines. The required sample size for a conclusive result was 1501. Z-curve crossing over the O’Brian-Fleming monitoring boundaries indicates that corticosteroids can decrease the length of hospital stay than the control group in severe community-acquired pneumonia patients.


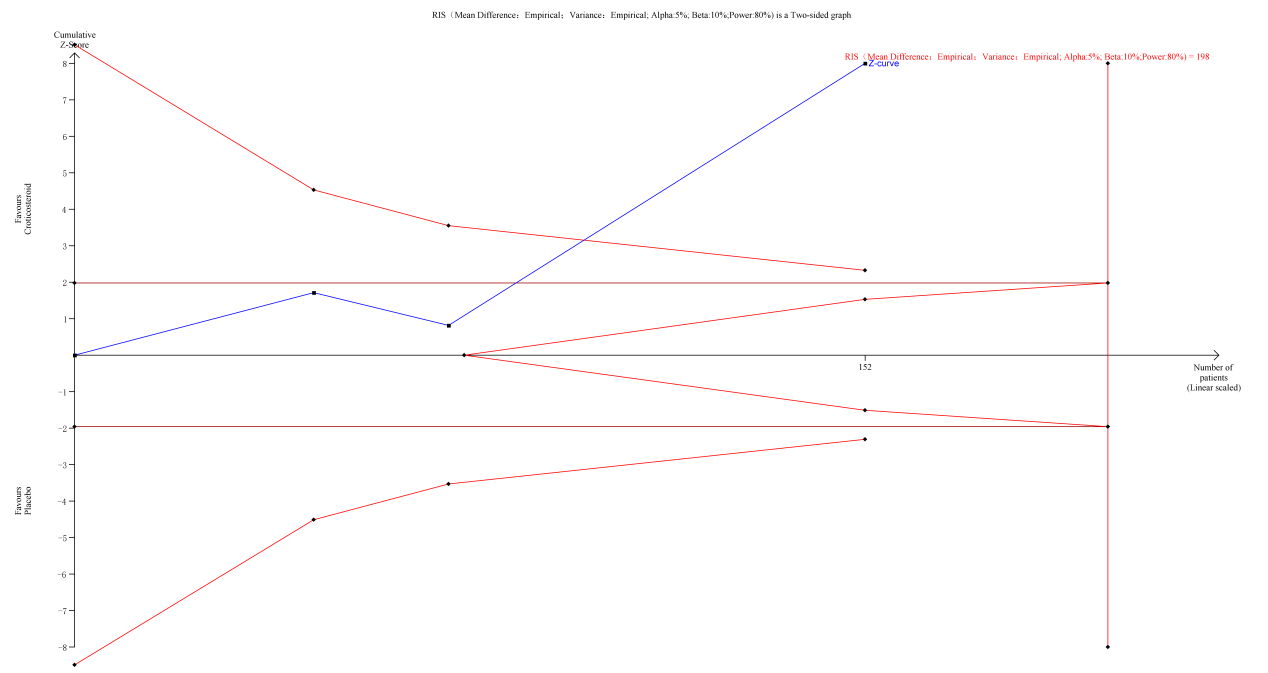
**Figure S10.Trial sequential analysis for the length of hospital stay in included trials receiving hydrocortisone therapy versus control group.** The blue line represents the cumulative Z-curve. The red line is the O’Brian-Fleming monitoring line (TSA) and the futility line. The purple lines are conventional P = 0.05 lines. The required sample size for a conclusive result was 198. Z-curve crossing over the O’Brian-Fleming monitoring boundaries indicates that hydrocortisone can decrease the length of hospital stay than the control group in severe community-acquired pneumonia patients.


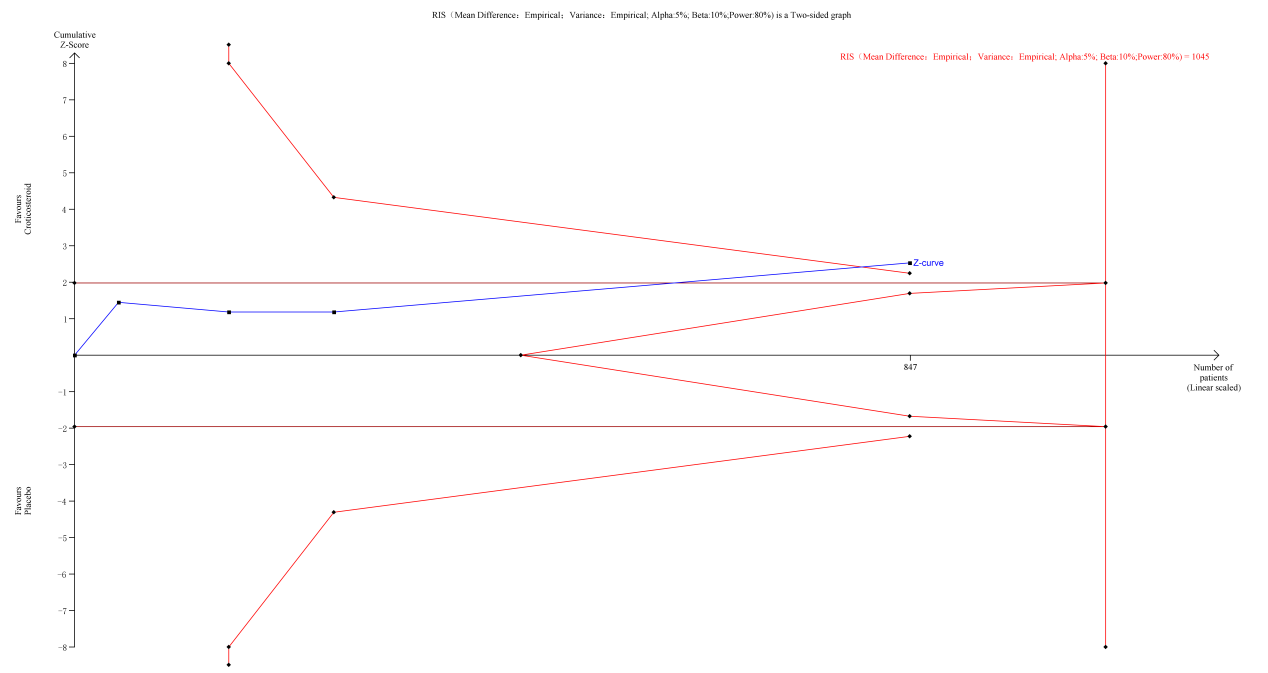


**Figure S11.Trial sequential analysis for the length of hospital stay in included trials receiving non-hydrocortisone therapy versus control group.** The blue line represents the cumulative Z-curve. The red line is the O’Brian-Fleming monitoring line (TSA) and the futility line. The purple lines are conventional P = 0.05 lines. The required sample size for a conclusive result was 1045. Z-curve crossing over the O’Brian-Fleming monitoring boundaries indicates that non-hydrocortisone can decrease the length of hospital stay than the control group in severe community-acquired pneumonia patients.


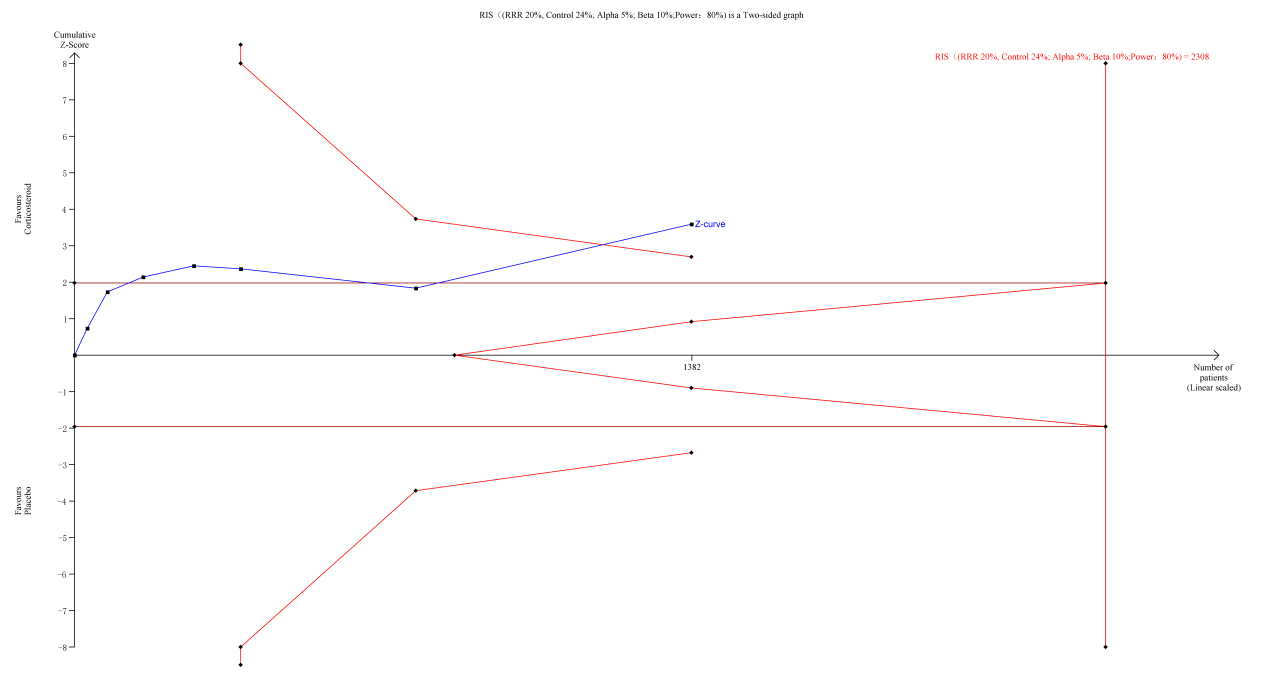


**Figure S12.Trial sequential analysis for the need for mechanical ventilation in included trials receiving corticosteroids therapy versus control group.** The blue line represents the cumulative Z-curve. The red line is the O’Brian-Fleming monitoring line (TSA) and the futility line. The purple lines are conventional P = .05 lines. The required sample size for a conclusive result was 2308. Z-curve crossing over the O’Brian-Fleming monitoring boundaries indicates that corticosteroids can decrease the length of mechanical ventilation than the control group in severe community-acquired pneumonia patients.

**
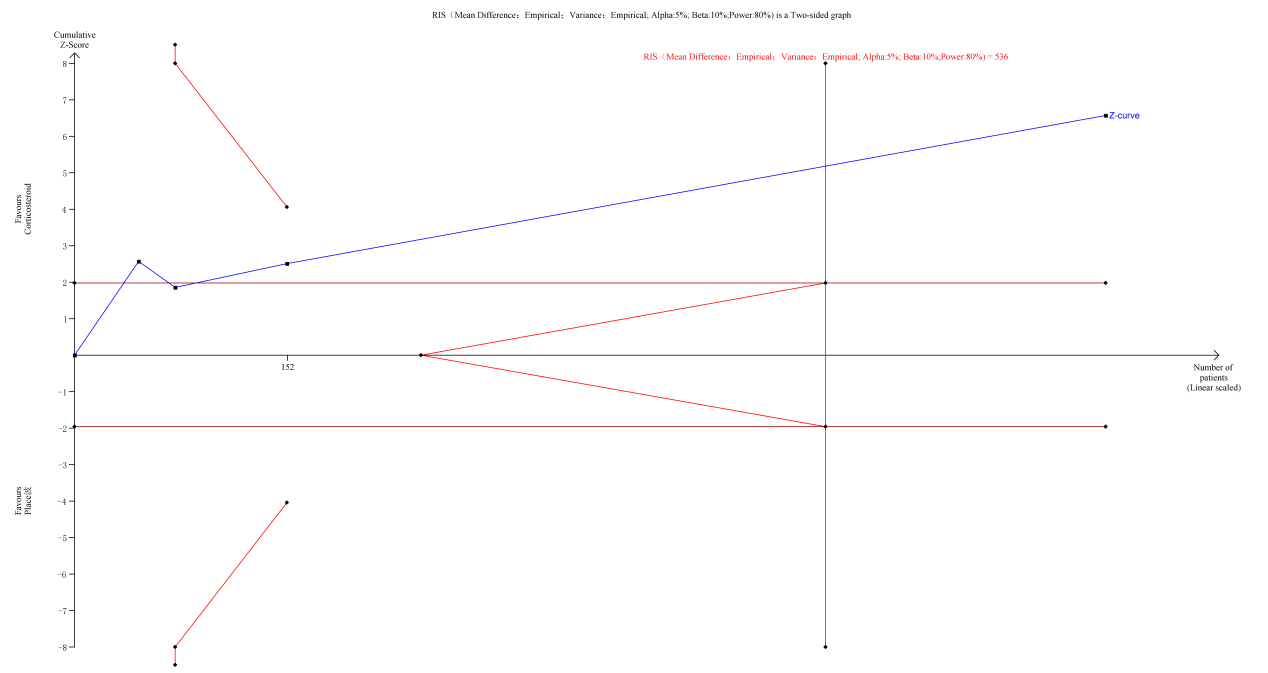
Figure S13. Trial sequential analysis for the mechanical ventilation time in included trials receiving corticosteroids therapy versus control group.** The blue line represents the cumulative Z-curve. The red line is the O’Brian-Fleming monitoring line (TSA) and the futility line. The purple lines are conventional P = .05 lines. The required sample size for a conclusive result was 536. Z-curve cross the traditional P = .05 lines and RIS, nor does it cross the O’Brian-Fleming monitoring line. There was no statistically significant difference in efficacy between the corticosteroids therapy and the control group for the mechanical ventilation time.

**
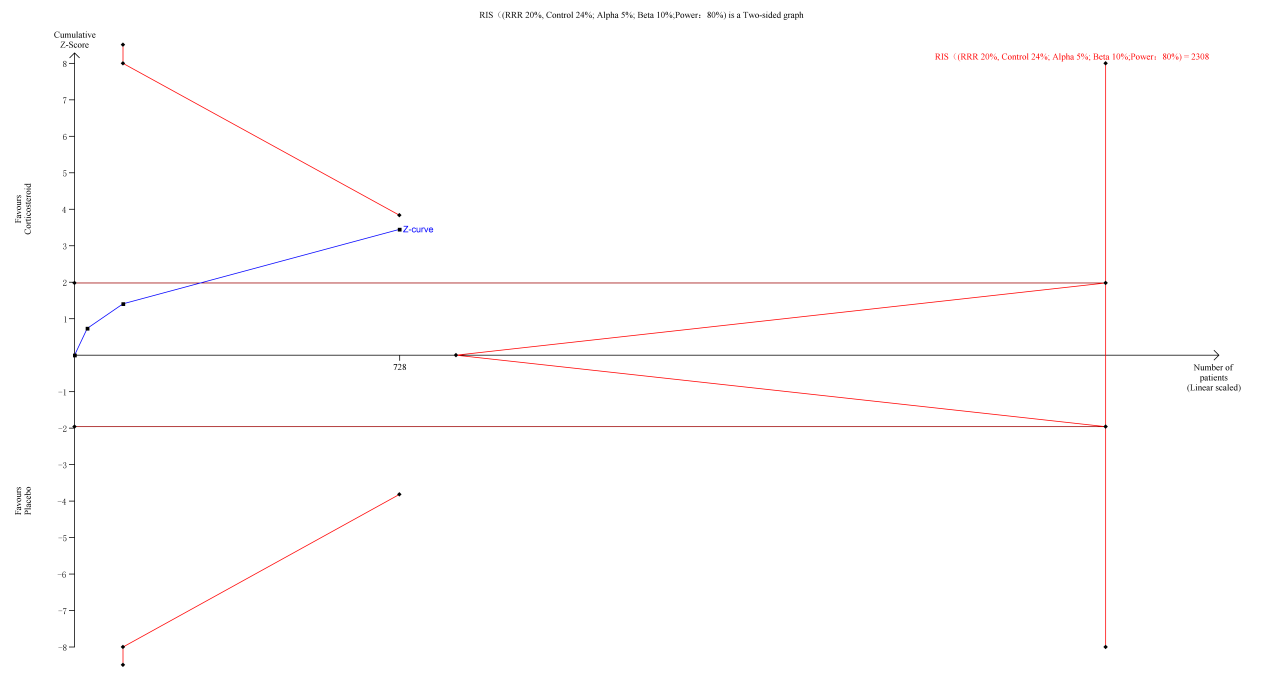
**

**Figure S14.Trial sequential analysis for the need for mechanical ventilation in included trials receiving hydrocortisone therapy versus control group.** The blue line represents the cumulative Z-curve. The red line is the O’Brian-Fleming monitoring line (TSA) and the futility line. The purple lines are conventional P = .05 lines. The required sample size for a conclusive result was 2308. Z-curve crossed conventional P = .05 lines , but did not reach O’Brian-Fleming monitoring line indicates that available results support hydrocortisone therapy more beneficial than the control group results but need further studies to avoid false positive results.

**
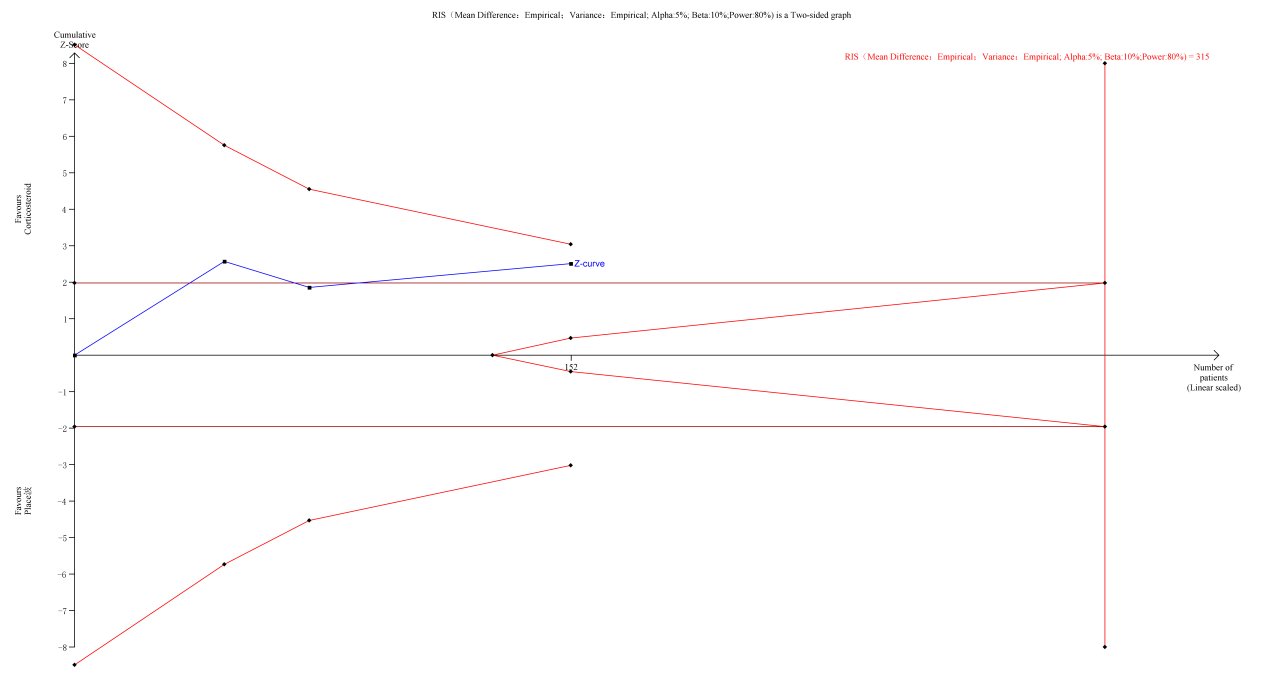
**

**Figure S15.Trial sequential analysis for the mechanical ventilation time in included trials receiving hydrocortisone therapy versus control group.** The blue line represents the cumulative Z-curve. The red line is the O’Brian-Fleming monitoring line (TSA) and the futility line. The purple lines are conventional P = .05 lines. The required sample size for a conclusive result was 315. The blue line represents the cumulative Z-curve. The red line is the O’Brian-Fleming monitoring line (TSA) and the futility line. Z-curve crossed conventional P = .05 lines , but did not reach O’Brian-Fleming monitoring line indicates that available results support hydrocortisone therapy more beneficial than the control group results but need further studies to avoid false positive results.

**
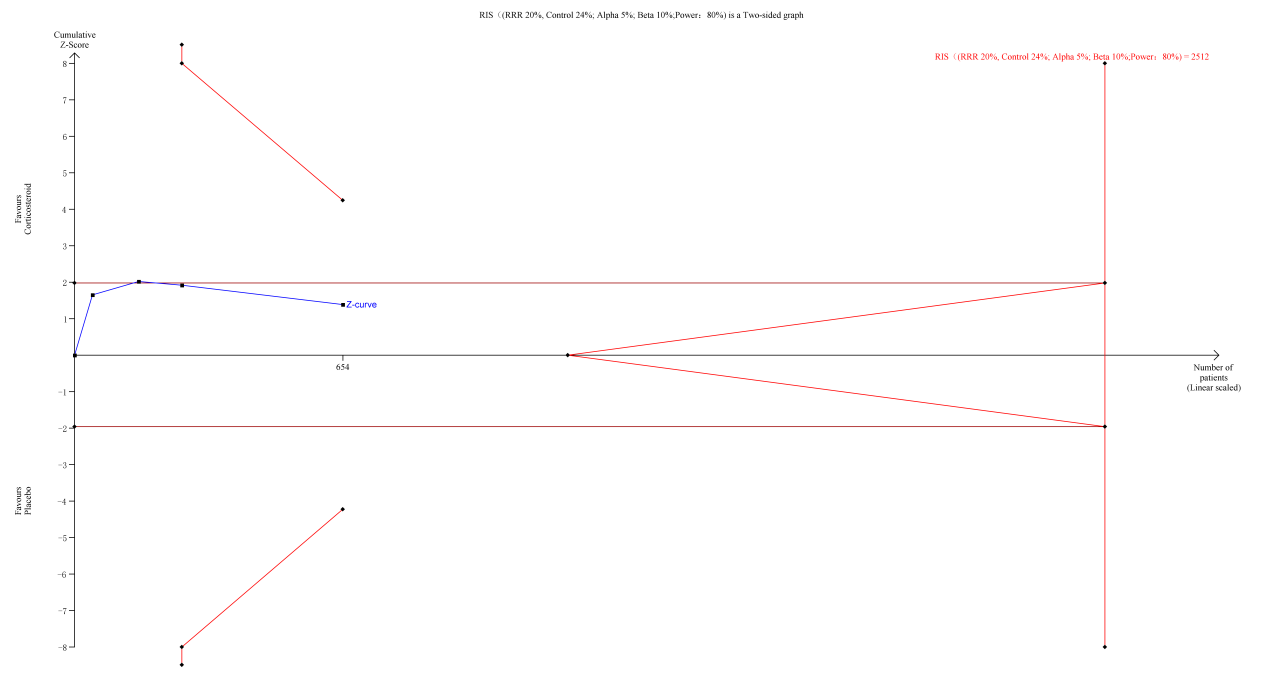
**

**Figure S16.Trial sequential analysis for the need for mechanical ventilation in included trials receiving non-hydrocortisone therapy versus control group.** The blue line represents the cumulative Z-curve. The red line is the O’Brian-Fleming monitoring line (TSA) and the futility line. The purple lines are conventional P = .05 lines. The required sample size for a conclusive result was 2512. Z-curve does not cross the traditional P = .05 lines, nor does it cross the O’Brian-Fleming monitoring line and did not reach the RIS. There was no statistically significant difference in efficacy between the non-hydrocortisone therapy and the control group.

**
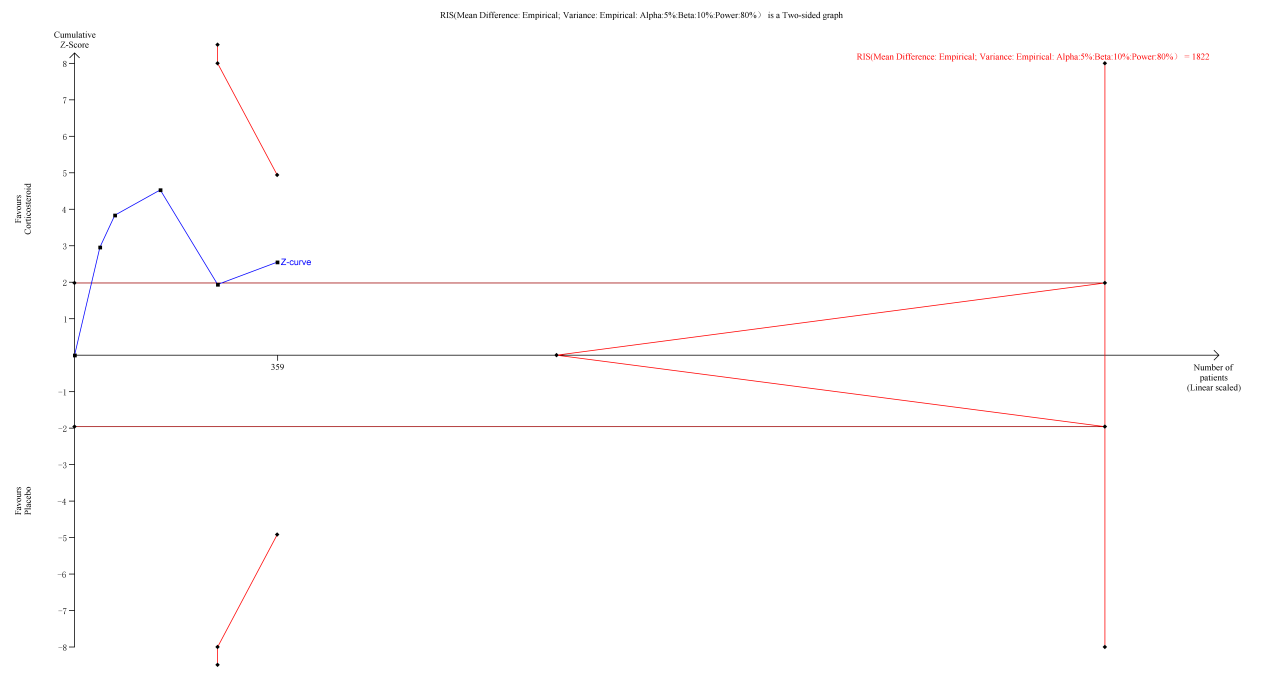
**

**Figure S17.Trial sequential analysis for the CRP levels in included trials receiving corticosteroids therapy versus control group.** The blue line represents the cumulative Z-curve. The red line is the O’Brian-Fleming monitoring line (TSA) and the futility line. The purple lines are conventional P = .05 lines. The required sample size for a conclusive result was 1822. Z-curve crossed conventional P = .05 lines , but did not reach O’Brian-Fleming monitoring line indicates that available results support corticosteroids therapy more beneficial than the control group results but need further studies to avoid false positive results.

**
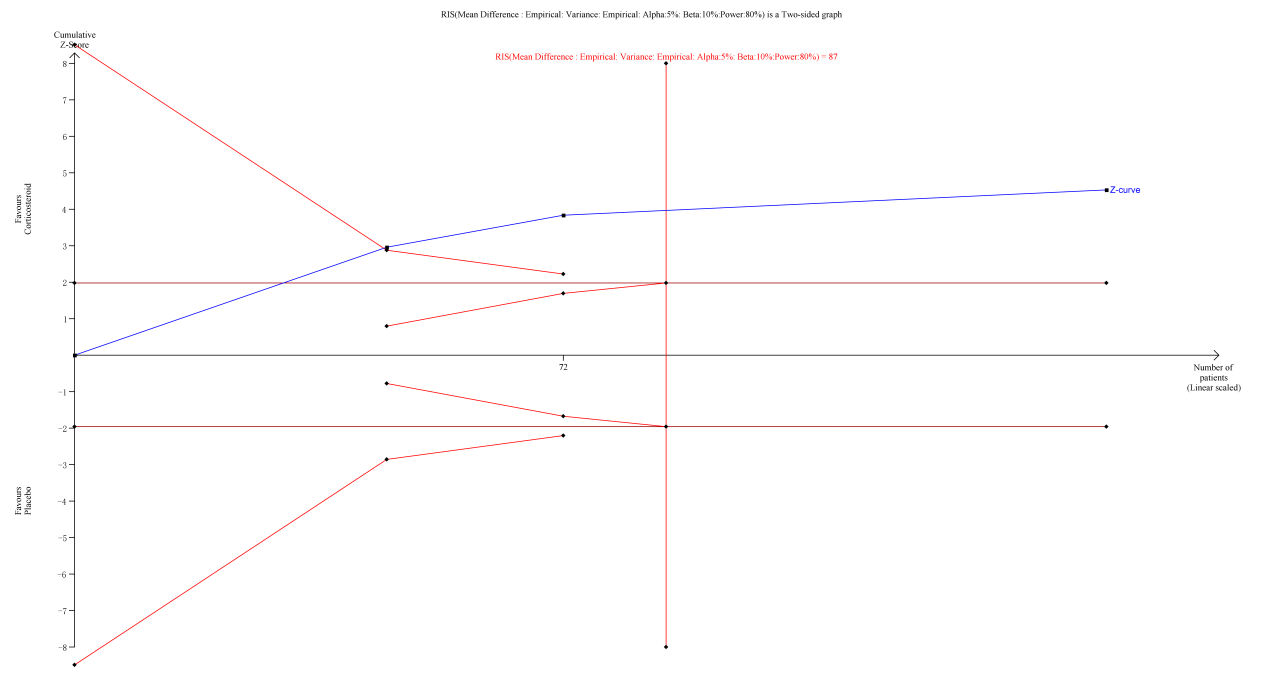
**

**Figure S18.Trial sequential analysis for the CRP levels in included trials receiving hydrocortisone therapy versus control group.** The blue line represents the cumulative Z-curve. The red line is the O’Brian-Fleming monitoring line (TSA) and the futility line. The purple lines are conventional P = .05 lines. The required sample size for a conclusive result was 87. Z-curve cross the traditional P = .05 lines, RIS, and the O’Brian-Fleming monitoring line which indicated that hydrocortisone therapy is more beneficial than control group in severe community-acquired pneumonia patients.
